# Identifying therapeutic drug targets using bidirectional effect genes

**DOI:** 10.1038/s41467-021-21843-8

**Published:** 2021-04-13

**Authors:** Karol Estrada, Steven Froelich, Arthur Wuster, Christopher R. Bauer, Teague Sterling, Wyatt T. Clark, Yuanbin Ru, Marena Trinidad, Hong Phuc Nguyen, Amanda R. Luu, Daniel J. Wendt, Gouri Yogalingam, Guoying Karen Yu, Jonathan H. LeBowitz, Lon R. Cardon

**Affiliations:** BioMarin Pharmaceutical Inc., Novato, CA USA

**Keywords:** Target identification, Rare variants

## Abstract

Prioritizing genes for translation to therapeutics for common diseases has been challenging. Here, we propose an approach to identify drug targets with high probability of success by focusing on genes with both gain of function (GoF) and loss of function (LoF) mutations associated with opposing effects on phenotype (Bidirectional Effect Selected Targets, BEST). We find 98 BEST genes for a variety of indications. Drugs targeting those genes are 3.8-fold more likely to be approved than non-BEST genes. We focus on five genes (*IGF1R, NPPC, NPR2, FGFR3*, and *SHOX*) with evidence for bidirectional effects on stature. Rare protein-altering variants in those genes result in significantly increased risk for idiopathic short stature (ISS) (OR = 2.75, *p* = 3.99 × 10^−8^). Finally, using functional experiments, we demonstrate that adding an exogenous CNP analog (encoded by *NPPC*) rescues the phenotype, thus validating its potential as a therapeutic treatment for ISS. Our results show the value of looking for bidirectional effects to identify and validate drug targets.

## Abstract (web summary)

Prioritising genes as potential drug targets is challenging and often unsuccessful once testing efficacy in humans. Here, the authors propose an approach to identifying drug targets that uses evidence from gain- or loss-of-function mutations associated with bidirectional effects on phenotypes.

## Introduction

Drug discovery has recently focused on target-based approaches to treat human disease instead of phenotypic screens. There are several ways in which potential targets are identified, the most common of which is the exploration of targets that have shown a mechanistic effect in either cell or animal models. The main disadvantage of these strategies is the high risk of failure when drug efficacy is tested in humans. Human genetic evidence for a target can improve the odds of success. Previous reports have suggested that a target with GWAS evidence has a 2-fold higher probability of successful clinical development compared to those targets with no evidence^1^. GWAS studies have produced thousands of hits for complex phenotypes^2^, leading to challenges in prioritizing the large number of potential targets for those most likely to be amenable to therapeutic intervention.

There are different approaches to prioritizing genetic targets^3–5^. The identification of multiple genetic variants in a single gene that associate with the outcome of interest (an allelic series) and mimic a dose-response curve can provide a direct indication that a promising target has been selected^5^. It can also help to quantify the magnitude of the required modulation needed to achieve efficacy, and even inform us of potential adverse events^5^. Such an allelic series may include risk variants, protective variants or both.

The presence of risk variants with large effects provides evidence for the key involvement of the gene in disease etiology. The presence of protective variants suggests that a therapeutic intervention that mimics the effect of those variants will be beneficial. The presence of protective variants in healthy individuals also provides evidence that an intervention mimicking the effect of those variants is less likely to have serious toxic effects, as otherwise those protective variants would not be prevalent. Moreover, if those protective variants are found in multiple unrelated individuals, instead of sporadic family members without disease, the likelihood that a drug mimicking their effect will be efficacious in a wide range of patients increases. Since the carriers of the protective variants likely have different polygenic risks and environmental exposures, their existence suggests that a treatment that mimics the beneficial effect of the variants will not be limited to patients with a monogenic condition caused by the same gene target. This is particularly relevant for developing treatments for polygenic diseases such as cardiovascular disease as exemplified by the use of *PCSK9* inhibitors to treat high lipid levels regardless of the genetic etiology as further explained further below^6^.

**Fig. 1:**
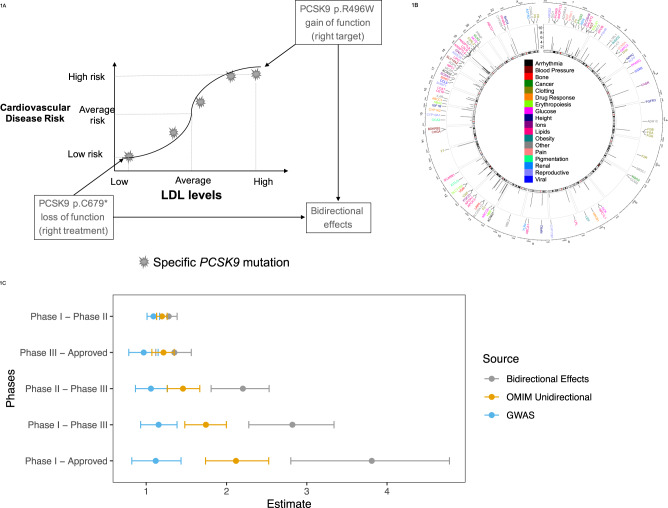
Generalizability of bidirectional effects for increasing probability of success on clinical trials. **A** Graphical representation of a gene (PCSK9) showing bidirectional effects on LDL and cardiovascular disease risk. **B** Circos plot of 98 genes with bidirectional phenotypic effects. From outside to inside: all autosomes and the X chromosome are depicted in a clockwise orientation. The genomic coordinates are shown for each gene that was identified to contain different variants with phenotypically opposing HGMD annotations. The panels with radial lines display the total number of HGMD variant entries (log base 2) that were categorized as bidirectional, for each gene. Each gene label is color coded based on the phenotype that displayed bidirectionality. **C** Estimated odds ratio for transitions between various clinical trial phases. We calculated the odds ratio for transition and 95% confidence interval, for the subset of target-indications with genetic support from GWAS, OMIM associations with unidirectional effects, and bidirectional effects curated from HGMD. Measure of center for the error bars represent the estimated odds ratios and the error bars represent the 95% confidence intervals. Bidirectional effect supported data is shown in gray, OMIM unidirectional support is shown in orange, and GWAS support is shown in blue. *n* = 26,884 target-indication pairs.

It has been proposed that large case–control studies (with over 25,000 cases) are required to have enough statistical power to identify genes with rare coding variants associated with modest risk for common complex diseases^7^. An alternative approach consists in identifying gain-of-function (GoF, missense variants that enhance or create protein activity) and loss-of-function (LoF, including Protein Truncating Variants or PTVs, i.e., stop-gained, splice site disrupting, frameshift, as well as missense variants that inactivate protein activity) variants affecting an intermediate phenotype related to the disease. For example, *PCSK9* GoF variants associate with higher LDL levels and higher cardiovascular disease (CVD) risk^8^. Conversely, *PCSK9* LoF have the opposite effect (Fig. 1A; lower LDL levels, lower CVD risk)^9^. A case–control study of 9524 subjects was required to demonstrate a significant effect on CVD^10^. This observation led to the therapeutic hypothesis that anti-PCSK9 neutralizing antibodies would lower LDL levels in all patients regardless of their *PCSK9* carrier status and also decrease risk for CVD. This strategy was eventually validated in successful Phase III randomized clinical trials^6,11^. Similarly, GoF and LoF variants with bidirectional effects on two members of the WNT pathway (*LRP5* and *SOST*)^12^ led to the creation of a new therapy for osteoporosis using antibodies against *SOST*^13^.

Height is an easily measured quantitative trait that has historically been used as a model to understand the genetic architecture of other complex traits and diseases. Human height is highly heritable (estimates range from *h*^2^ = 70–90%)^14^. At the low end of the height distribution, idiopathic short stature (ISS, defined as having a height at least two standard deviations (SDs) below the mean)^15^ affects ~2% of the general population. Mutations in several genes have been described in studies of familial ISS as well as in cohorts of ISS patients^16^.

Mendelian forms of extreme short stature (height at least 5 SDs below the mean), such as Achondroplasia, Acromesomelic dysplasia type Maroteaux (AMDM), and Osteogenesis Imperfecta type III, are less frequent (1/15,000, 1/1 million, unknown respectively)^17–19^. There is no approved treatment for these extreme forms of short stature. However, a C-type natriuretic peptide (CNP) analog is being investigated in a phase III Randomized Clinical Trial for Achondroplasia^20^. And while growth hormone has been used for ISS, response to treatment is variable^21^. Thousands of loci have been associated with height using GWAS^22–27^. Linking those association signals to genes, and those genes to mechanisms that may ultimately yield a new drug target has been challenging despite large scale efforts to map regulatory regions^28,29^.

Here, we propose that genes defined by allelic series with both risk and protective variants of large effect (Fig. 1A; Bidirectional Effect Selected Targets (BEST)) are preferable as drug targets compared to those limited to one or the other. To test this hypothesis, we identify 98 genes with bidirectional effects on reported human traits or diseases. Then we show that a wide variety of drugs targeting a BEST gene are more likely to advance in the approval process. We chose height as a tractable model of a complex trait to further test our hypotheses and provide a mechanistic understanding. We prioritize height genes to identify the subset most likely to be good therapeutic targets by selecting those exhibiting bidirectional effects on height. We functionally validate genetic variants and compare the predicted effect of these coding variants with their functional impact at a cellular level as well as with a clinical readout. We then validate the use of a therapeutic approach in a cell model to rescue the molecular phenotype.

## Results

### Bidirectional effect selected targets

We assessed the generalizability of bidirectional effects as predictors of clinical trial success on a diverse set of human diseases and traits. To address this, we identified 98 genes in HGMD that showed evidence of bidirectional effects for different gene variants (Methods section, Fig. 1B and Supplementary Data 1). There were multiple diseases where potential bidirectional genes were identified. The phenotypes with the highest number of bidirectional genes were: lipids, clotting, reproduction, height, and glucose (19, 12, 5, 5, and 5 respectively). These five groups accounted for 45% of all bidirectional gene – phenotype pairs in this database (Fig. 1B). We then calculated the odds of clinical trial approval for BEST target-indication pairs using a database of clinical trial outcomes^30^. Finally, we compared those odds to the odds of targets with purely unidirectional evidence from OMIM and targets with evidence from GWAS (Methods section, Supplementary Data 2). We found that overall, the BEST target-indications that have reached Phase I have higher odds of successful transition to Approval as compared to other non-BEST target-indications (maximum RR = 3.81 95% CI [2.81–4.74], Supplementary Fig. 1, Supplementary Data 3, and Fig. 1C). Importantly, those odds were significantly higher than that of other sources of genetic evidence (Fig. 1C, RR = 2.12 95% CI [1.73–2.52], RR = 1.12 95% CI [0.82–1.46] for OMIM unidirectional and GWAS targets respectively). BEST target-indications showed significant improvement in the odds of successful transition at all steps of the clinical development as compared to non-BEST target-indications (Fig. 1C). Interestingly, the phase where BEST target-indications showed the strongest effect was the transition from Phase II to Phase III (RR = 1.46 95% CI [1.25–1.66]), a phase that is generally used as proof of concept for initial clinical efficacy. Sensitivity analyses confirmed that these observations were not confined to a single disease category biasing the global signal and thus support that BEST genes may have better probabilities of success in clinical trials across multiple disease indications (see Methods section, Supplementary Figs. 2–4.

### Bidirectional growth genes

To further understand bidirectional genes that are regulators of growth, we analyzed the intersections of five gene lists (GWAS, HGMD short, HGMD tall, OMIM short, OMIM tall; see Methods). Growth regulators would be the most likely to contain rare coding mutations with bidirectional effects (i.e. short stature or skeletal dysplasia AND tall stature or overgrowth). There were 47 genes annotated with at least one pathogenic variant reported in the literature to cause “short stature”. Only 20 genes were annotated as tall stature or overgrowth genes (Supplementary Figs. 5 and 6 and Supplementary Data 4 and 5). Secondly, we used a manually curated list of 258 OMIM genes (248 short, 20 tall) which was created using the keywords: short stature, overgrowth, skeletal dysplasia, and brachydactyly^24^. Third, we looked at the intersection of these four lists with the list of genes from GWAS (Supplementary Fig. 6 and Supplementary Data 5). We found at the intersection of these five lists contained three genes known to be associated with height (Set 1: *IGF1R*, *NPPC*, and *NPR2*). Two additional genes were present in the intersection of all lists except GWAS (Set 2: *FGFR3* and *SHOX*). *FGFR3* was not in Set 1 as it was not the closest gene in the region of any GWA signal and *SHOX* was not tested in the GWAS study as it is located on the X-chromosome (Supplementary Figs. 7–10). The *NPPC*/*NPR2* and the *IGF1R*/growth hormone pathways have been implicated in GWAS of height^25,26^.

### Gene set analysis

Literature on extreme phenotypes such as short or tall stature is susceptible to publication bias. A randomly ascertained study population is required to obtain an unbiased assessment of the effects of rare coding variation of these genes on height on the general population. For that purpose, we used exome sequence data from 33,204 white British individuals from the UK Biobank project of which 572 (1.67%) reached the criterion of ISS (Supplementary Table 1; see Methods section). After quality control, we identified 245,083 rare (allele frequency (AF) < 0.01%) missense and PTV variants in 1964 genes with at least one rare protein coding variant. In all, 1918 genes with a mean cumulative allele count (AC) ≥20 remained after removing those with a cumulative AC < 20 (see Methods section).

To evaluate if any of the annotations (GWAS signal adjacent, short stature, tall stature, both short and tall, etc.) had a significant association with height, gene set analysis was performed using the combined effect of any rare protein modifying variants using the SNP-set (Sequence) Kernel Association Test (SKAT)^31^. There were 32 possible intersections of the 5 annotations described above and 25 of those contained at least one gene (Supplementary Fig. 6 and Supplementary Table 2). Of these, surprisingly, only the two aforementioned gene sets (Set 1 and Set 2) showed significant association with height. They both contained bidirectional effects as reported by both HGMD and OMIM, namely: Set 1: *NPR2*, *NPPC*, *IGF1R* (*p* = 6.82 × 10^−7^) and Set 2: *FGFR3 and SHOX* (p = 1.75 × 10^−5^; Table 1, Supplementary Fig. 6, and Supplementary Table 2). Given that both sets of genes share the property of having bidirectional effects on height, we combined all 607 independent rare protein-altering variants for the five genes in Set 1 or Set 2 using burden tests. Mutations in this group of five genes significantly decreased height (*β* = −0.20, *p* = 4.04 × 10^−11^) and significantly increased risk for ISS (OR = 2.75, 95% CI [1.92–3.96], *p* = 3.99 × 10^−8^). Interestingly, this set of bidirectional genes has a much stronger risk for ISS as compared to the sets of genes reported for solely short stature or tall stature (Supplementary Fig. 11). Restricting the test to rare PTV variants resulted in an association with a larger decrease in height (Table 1, *β* = −0.85, *p* = 2.49 × 10^−6^). Of the 607 variants, 158 were singletons. Also, we observed that 192/607 (31.63%) mutations were not reported in any of the public human databases and thus may represent novel variants, some of which may be pathogenic (Supplementary Data 6).

**Table 1:** Individual gene association statistics for Height and Idiopathic Short Status (ISS, height < -2 SD below the mean) on rare protein-altering or PTV variants alone.

Gene set/gene	Orthogonal evidence			Burden height (protein-altering)			Burden height (PTV)			Burden Idiopathic Short Stature (ISS) (Protein-altering)					
	Human	Mice	Cell	AC	Effect	*P*-value	AC	Effect	*P-* value	AC case	AC ctr	Cum AF case (%)	Cum AF ctr (%)	OR 95%CI	*P-*value
Set 1															
* IGF1R*				264	−0.20	**0.001**	7	−0.76	0.042	12	252	2.0	0.7	2.85 [1.48–5.47]	**0.002**
* NPPC*				32	0.02	0.895	2	−1.43	0.042	2	30	0.3	0.1	4.83 [0.99–23.65]	0.052
* NPR2*				237	−0.26	**5.38** **×** **10**^**−5**^	12	−0.97	**0.001**	12	225	2.0	0.7	3.31 [1.68–6.51]	**0.001**
Set 2															
* FGFR3*				321	−0.19	**8.27** **×** **10**^**−4**^	7	−0.43	0.252	11	310	1.8	0.9	2.2 [1.13–4.28]	0.020
* SHOX*				83	−0.32	**0.003**	2	−1.36	0.052	4	79	0.7	0.2	3.34 [1.08–10.31]	0.036
Set 1 + Set 2				**937**	−0.20	**4.04** **×** **10**^**−11**^	30	−0.85	**2.49** **×** **10**^**−6**^	41	896	6.7	2.7	2.75 [1.92–3.96]	**3.99** **×** **10**^**−8**^

Bold *P*-values represent significant associations after adjusting for five tests.

Green and Red dots on the orthogonal evidence column reflects the presence of gain-of-function and loss-of-function mutations in the respective model with corresponding evidence of effect on growth (for more details, see Supplementary Table 3). *P*-values calculated from linear or logistic regression analysis for quantitative and discrete traits respectively using a two-sided test without adjustment for multiple testing.

*AC* allele count, *PTV* protein truncating variant, *Effect* effect estimate from regression, *P* nominal *P*-value estimate.

### Associated height genes

While most of these mutations are too rare to be significantly associated individually, each of the five associated genes (*FGFR3, IGF1R, NPPC, NPR2*, and *SHOX*) were nominally (*P* < 0.05) associated with height when considered individually, reflecting that no single gene was driving the gene set signal (Table 1, Methods section). The directions of effect was consistent with PTV variants in *NPR2* having a Bonferroni-adjusted significant association with shorter stature (*β* = −0.97, *p* = 0.001) and nominally significant associations in the same direction for *IGF1R* and *NPPC*. Rare missense variants were nominally associated with shorter height (*β* = −0.22, *p* = 0.002; *β* = −0.18, *p* = 0.003; and *β* = −0.18, *p* = 0.001) for *NPR2*, *IGF1R* and *FGFR3*, respectively. Combined PTV and missense variants in *NPR2* and *IGF1R* were also nominally associated with increased risk for ISS (OR = 3.31, *p* = 0.001, OR = 2.85, *p* = 0.002, respectively; Table 1).

### Polygenic scores

**Fig. 2:**
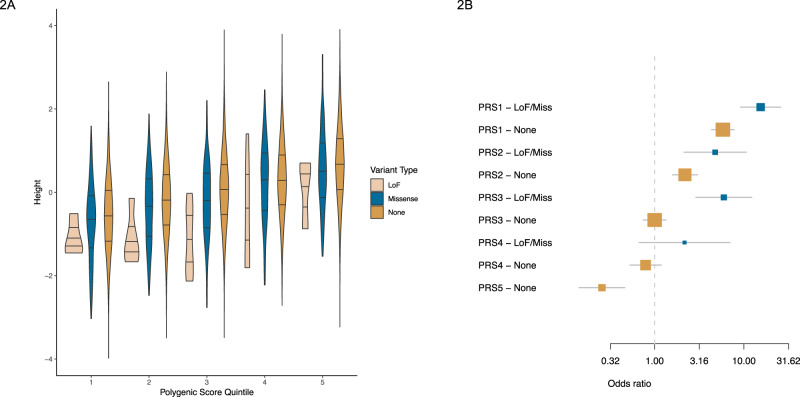
Effect of rare coding variants on bidirectional effect genes for height. **A** Combined effect of PS and rare coding variants on height as a quantitative trait. Samples were divided in five groups based on their PS. Horizonal lines in violin plots represent the 25%, 50% and 75% percentile of height. Samples were grouped by carrying status of missense (blue), loss of function (pink), or none (orange) in any of the five core genes. **B** Combined effect of PS and rare coding variants on odds ratios for “idiopathic short stature” or ISS. Odds for ISS using non-carriers of *NPPC, NPR2, FGFR3, SHOX*, and *IGF1R* with PS = 3 as reference vs the other PS groups. Box colors represent: orange, non-carriers of LoF/missense variants in the five genes; blue, carriers of LoF/missense variants in the five genes. Measure of center for the error bars represent the estimated odds ratios and error bars represent 95% confidence intervals. Cases *n* = 572; control *n* = 33,659.

The polygenic nature of complex traits suggests that the combined effect of thousands of variants may be an independent risk factor to rare variants with strong effects possibly modifying the overall risk. To test this hypothesis, we calculated polygenic scores (PS) for sex-adjusted height *Z*-scores using the largest published GWAS meta-analysis for height that did not include any samples from the UK Biobank project (see Methods section)^24^. We divided our cohort into five equally sized (*n* = 6824) PS quintiles (PS 1 being the lowest predicted height, PS 5 the tallest predicted height). As expected, there was a dose-dependent relationship between increasing PS score and mean height (*β* = 0.30 per each PS quintile increase; Fig. 2A). Carriers of PTVs in the five genes were consistently shorter than non-carriers across the five different PS backgrounds (Fig. 2A and Supplementary Figs. 12–16). Our data suggest that the combined effect of PS and rare protein variants is consistent with an additive model: polygenic effects modulated height in both carriers and non-carriers. The mean effect of having a rare protein coding variant in any of the five genes was similar across PS subgroups (*I*^2^ = 30%, *P*_het_ = 0.21; *I*^2^ = 0, *P*_het_ = 0.62 for missense and LoF variants respectively; Fig. 2A, Supplementary Figs. 17–23). We then calculated the risk for ISS across PS groups using individuals at PS3 who lacked mutations in the five genes as a reference. Non-carriers of the lowest PS group had increased risk for ISS and non-carriers of the highest PS group had decreased risk for ISS (Fig. 2B; OR = 5.81, *p* = 2.74 × 10^−32^; OR = 0.25, *p* = 6.09 × 10^−6^ for PS 1 and PS 5 respectively). We then evaluated the effect of rare coding variants of the five genes for ISS stratified by PS group. Carriers of rare coding variants in any of the five genes were at increased risk for ISS in the first three quintiles (OR = 15.39, *p* = 4.44 × 10^−25^; OR = 4.76, *p* = 1.34 × 10^−4^; OR = 5.96, *p* = 1.21 × 10^−6^; Fig. 2B). Carriers were not at significantly increased risk for ISS in the fourth quintile (OR = 2.16, *p* = 0.20). There were not enough ISS individuals in the highest PS group (14 ISS for PS 5, all of them were non-carriers).

### Functional assessment of protein coding variants

**Fig. 3:**
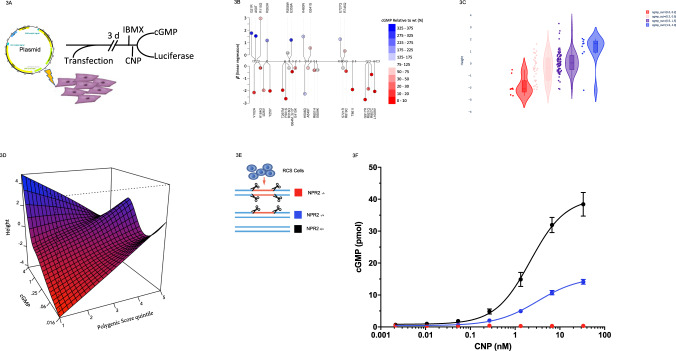
Functional effects of NPR2 protein coding variants. **A** Schematic for evaluation of missense variants. The bicistronic plasmid with a CMV promoter driving production of NPR2 variants and an EF1a promoter driving production of red firefly luciferase was transfected into HEK293T cells. Three days post transfection cells were treated with IBMX, incubated with CNP, and analyzed for luciferase and cGMP activity. cGMP activity was normalized of variants relative to luciferase, *n* = 3 biologically independent samples. **B** Diagram of NPR2 protein-altering variants identified in the UKBiobank study. *X*-axis indicates genomic position. *Y*-axis indicates effect size (*β*) of a linear regression of that variant on height in standard deviations. Each circle represents a different variant with the number of carriers inside the circle, amino acid change of the variant is labeled at the end of the circle. Color code indicates cGMP activity of variants relative to luciferase. Source data are provided in Supplementary Table 4. **C** Violin plots representing height of carriers of the NPR2 variants tested in functional analyses. Box-plots inside violin plots represent the median and inter-quantile range of height in the group. Whiskers are 95% CI. Color categories represent binned cGMP functional readout. Source data are provided in Supplementary Table 4. **D** Predicted measured height (Z-transformed) as a function of their PS and the percent of wild-type cGMP observed for their *NPR2* variant. The color maps to height (blue = highest, red=lowest). Source data are provided in Supplementary Table 5. **E** Schematic for generating NPR2 deficient cells using CRISPR/Cas9. **F** cGMP dose response to CNP treatment with *NPR2* knockout (red), heterozygous knockout (blue), or wild-type (black) cells, *n* = 3 biological independent samples; error bars represent mean ± s.d. Source data are provided as Source Data file.

Binding of C-type natriuretic peptide (CNP, encoded by the *NPPC* gene which was also on the list of genes with bidirectional effects, Table 1 and Supplementary Data 6) to its receptor (*NPR2)*, triggers endochondral and skeletal growth via cGMP production^32^. In order to explore the effect of gain of function mutations in *NPR2* on height, which should mimic the effect of adding exogenous CNP, we assessed the function of multiple missense mutations across the full spectrum of NPR2 activity. We selected 39 variants for functional analysis: 9 *NPR2* protein-altering mutations that were present in UKBiobank ISS individuals, 7 NPR2 missense mutations present in UKBiobank individuals with height >1.2 SD, 5 *NPR2* variants in individuals with a mean height around 0 (in SDs), and 4 additional *NPR2* mutations with higher carrier number to complement the allelic series. Additionally, we tested 13 *NPR2* mutations that were reported in the literature for ISS and/or a more severe recessive form of short stature (Methods section and Supplementary Table 4). To distinguish functional vs. neutral *NPR2* missense variants, we performed a series of experiments in cellular models (Methods section and Fig. 3A).

Constructs were tested in triplicate and the mean and SD of the functional readout (cGMP) was reported (Methods section and Supplementary Table 4). We first confirmed the dynamic range of the assay: 10 out of 11 previously reported pathogenic variants had mean cGMP < 0.20 relative to wildtype (wt) and 2 out of 3 previously reported gain of function variants showed a mean cGMP > 1 relative to wt (Supplementary Table 4). From the list of ISS patients found in our study, 10 out of 12 tested mutations were confirmed as low activity (mean cGMP < 0.2 relative to wt). (Fig. 3B and Supplementary Table 4). There was a significant association with increasing cGMP levels and increasing height (*β* = 0.91 per 100% change in cGMP relative to wt, *p* = 2.7 × 10^−7^; Fig. 3C). These assay data served as a better classifier for height than a predictor (combined annotation dependent depletion (CADD))^33^, (*β* = −0.02, *p* = 0.04 for CADD unit change score).

One interesting outlier was identified carrying the *NPR2* variant p.R110G. While this variant showed a modest loss of function in our experiments (cGMP = 0.70), the individual carrying it was very tall (height *z*-score = 3.10). A closer examination of this individual revealed that they were in the 99.85th percentile of the PS for height (Supplementary Fig. 24). This prompted us to examine PS groups in *NPR2* carriers of the variants we functionally tested. We grouped *NPR2* carriers by their PS group and their residual *NPR2* activity. Despite having a small number of individuals in each group, as shown before there was an additive relationship between PS and *NPR2* functional variants on height (Fig. 3D and Supplementary Table 5). This additive relationship was consistent with the combination of multiple risk factors of PS and rare variants with strong effects.

### Phenotype rescue

In order to test whether a CNP-analog can rescue a LoF mutation in *NPR2*, we examined the response to CNP of cells engineered to have heterozygous or homozygous LoF mutations. We measured the amount of cGMP produced by CNP stimulation in CRISPR/Cas9-mediated knockout chondrocytes (Methods section and Fig. 3E). cGMP production by NPR2 triggers a signaling cascade via activation of protein kinase cGMP-dependent 2 (*PRKG2*). Previous activation data reports cGMP EC_50_ in the range of 40–360 nM for activation of PRKG2^27,34,35^. In the heterozygous *NPR2* knockout cells, a CNP dose >0.163 nM is able to achieve an intracellular concentration exceeding the EC_50_ range for PRKG2 activation (Fig. 3F). Whereas, in wild-type cells a CNP dose of 0.040 nM is able to achieve the same cGMP concentration. These results demonstrate that CNP supplementation can achieve the cGMP levels necessary for PRKG2 activation and growth in cells with loss-of-function mutations of *NPR2*.

## Discussion

We have shown that drug targets with bidirectional effects have increased probability of success (nearly 4-fold) in clinical trials. Importantly, this increase goes above and beyond the increased probability for targets with unidirectional human genetic evidence (2-fold). Our results have important implications not only for drug discovery but also for drug repurposing. The presence of genes driving disease in the general population suggests the possibility of tailoring therapies to the small fraction of individuals with gene defects. Also, the fact that such genes have allelic series with bidirectional effects and additive effects across PS groups, suggests that such tailored therapies may be effective for any patient irrespective of the genetic lesion. Accordingly, our results show that treatments based on BEST genes were approved for an indication regardless of genetic cause, for example, treatments for hypercholesterolemia, hyperparathyroidism, dwarfism, gout, and breast neoplasms (Supplementary Data 2). While our results show bidirectionality in a wide range of endpoints, the limited number of bidirectional genes (98) limits our ability to conclusively demonstrate that our observations will hold for other clinical endpoints for which we still do not have sufficient genetic findings. Many of the approved drugs targeting bidirectional genes are linked to an approved biomarker of a clinical endpoint. Approaches to better identify bidirectional genes in the absence and/or incomplete knowledge of the relevant biomarkers behind a disease warrants future research. Also, better understanding of the safety vs efficacy components of BEST genes is an area of potential future research.

We further identified and validated the genes most likely to be drug targets for ISS by showing mutation bidirectionality (GoF and LoF). We found a set of five height genes with bidirectional effects and demonstrated that variants in these have large effects on height. We also characterized the molecular phenotypes of 39 mutations in one of these genes and show that they correspond to the observed phenotype across different height PS backgrounds. Height has been used as model for common complex traits and diseases and its genetics are characterized by a highly polygenic architecture. Despite this polygenic nature, there are critical genes where single LoF/GoF variants have large effects with magnitudes approaching the aggregated effects of thousands of common variants with small effects (as calculated by PS). We provided evidence that genetic insults to these five genes contribute not only to rare skeletal malformations (Table 1 and Supplementary Data 7), but also to common forms of short stature. Additional evidence from both human and animal models support the notion that the *NPPC*-*NPR2*-*FGFR3*-*SHOX* pathway is a key modulator of growth (Supplementary Table 3). In the same way, *IGF1R*, a key member of the growth hormone receptor pathway, points toward a second group of genes that would include IGF-I which is currently a short stature therapy for Growth Hormone non-responsive patients^36^.

Entire gene deletions and/or mutations causing loss of protein function in *SHOX*, *IGF1R*, *NPPC*, and *NPR2* have been reported in familial short stature with various degrees of severity (Table 1 and Supplementary Table 3)^18,37–40^. Conversely, duplications, deletions of repressor regions, translocations, and missense mutations leading to GoF in these genes were reported in individuals with tall stature or overgrowth^41–45^. GoF mutations are hard to identify from in-silico predictions and usually require functional validation. GoF mutations in *FGFR3* cause Achondroplasia, the most common form of dwarfism^46^. In the opposite direction, LoF mutations in *FGFR3* cause overgrowth^46^. For both *NPPC* and *IGF1R*, only large translocations and duplications have been reported with the overgrowth phenotype. In contrast, at least four independent *NPR2* missense mutations have been reported to be GoF^39,43,44,47^. Importantly, mutations in these genes in animal and cell models also show bidirectional effects that are consistent with the directions of effects observed in humans (Supplementary Table 3).

The additive effects of common genetic variation (i.e. PS), predicted 20.1% of the variance in height in our dataset. These effects appeared to have similar magnitudes on the carriers of rare coding variation of genes as compared to non-carriers. This observation indicates that polygenicity may be a strong contributor in the differences in penetrance of rare pathogenic variants (especially in models of haploinsufficiency such as the ones described here). Supporting this idea, we saw that two of eight *NPR2* variant carriers with low NPR2 activity had a low-normal height. One of them was in the 4th PS quintile and the other was in the 5th PS quintile, the remaining six were in quintiles 1–3 (Fig. 3D and Supplementary Table 5). This data suggests that most ISS individuals possessing mutations in *NPR2* may also have a polygenic background that made them more susceptible to the pathogenic effect of losing *NPR2* activity. This observation could be interpreted as support for the omnigenic model^48,49^, where core genes would define a module that when disrupted, has large effects. Similarly, the omnigenic model predicts that the effect of core genes is modulated by multiple weaker common genetic variants driving regulatory networks. Our data show that the polygenic background can indeed modulate the effect of rare mutations with large effects, consistent with a simple additive model observed in sex chromosome aneuploidies that affect height^50^.

It is possible that other genes will appear as bidirectional modulators of height as sample sizes increases. Our study was 63% powered to identify a gene set association that explained the proportion of variance we observed in the set of five genes we describe. A follow-up study would require over 400,000 samples to have 80% power to identify individual genes at exome-wide significance levels (see Methods section). We also simply used the closest gene to a GWAS signal to annotate height genes. Other methods to map variants to genes are available and strategies such as co-localization may be useful to improve the detection of BEST genes using a combination of exome and GWAS data.

Here we demonstrate that carriers of variants in any of the five genes are at ~3-fold increased risk for ISS and account for ~6% of the total ISS population. Loss-of-function mutations in *NPR2* are responsible for dwarfism in mice and a lack of an intracellular cGMP response to CNP in cultured chondrocytes^51^. Here we showed dose-dependent rescue of *NPR2* signaling in a cell model of *NPR2* haploinsufficiency after adding exogenous CNP. While technological innovation is required to design novel cell models that allows studying proliferation of a chondrocyte model, our results support the idea that these CNP-based treatments could be effective in NPR2 haploinsufficient patient populations if the right dose can be delivered. Further, our results show a significant correlation between cGMP levels and height in carriers of variants in *NPR2* within the general population. More importantly, multiple *NPR2* gain-of-function carriers with different polygenic backgrounds were taller than non-carriers, further suggesting that targeting this receptor with CNP analogs could be an effective therapy for all ISS individuals. This is also supported by animal model studies showing that wild-type rhesus macaques exhibit increased growth velocity when treated with CNP^52^. These observations support the notion that drugs with BEST genes are more likely to be approved because they have molecular evidence that targeting that gene will have an effect in the underlying cellular phenotype into the direction that reverses the disease pathology.

A recent publication documented the height trajectory of seven *NPR2* heterozygote carriers from two families with *NPR2* pathogenic variants. These *NPR2* carriers display a pattern of progressive decline of age-adjusted height *z*-scores eventually leading to severe short stature^53^. While growth-hormone is approved in the broad ISS category, conflicting lines of evidence from small clinical studies on *NPR2* heterozygote patients are hard to interpret^53–55^. An open-label phase 2 study is currently recruiting *NPR2* carriers and other forms of genetic short stature and will be testing a CNP-analog as a potential treatment for their growth deficit (ClinicalTrials.Gov number NCT04219007).

In summary, we highlight a strategy to identify drug targets with higher probability of success. We tested the approach by identifying drug targets for short stature by leveraging a combination of human genetic databases and validation using large-scale exome sequencing studies followed by functional studies.

## Methods

### Bidirectional screen for broader indications

The HGMD allmut table v2016.4 was used as the source for associations to identify bidirectional effect associated genes. The table was grouped by each combination of gene and disease. Any genes with less than two unique disease associations were excluded from further analysis. The remaining dataset contained 23,754 distinct gene-disease pairs between 4427 genes and 12,302 diseases. After removing the gene names to avoid a potential source of bias, this table was manually curated to identify genes with at least two associations that appeared to have different directions of effect on a common underlying phenotype. For example, *PSCK9* was identified as a bidirectional effect gene due to associations with “high LDL cholesterol” and “low LDL cholesterol”. *KCNJ2* was identified as a bidirectional effect gene due to associations with “long QT syndrome” and “short QT syndrome”. We also required that the disease field represent an actual disease or disease associated endophenotype such as a blood metabolite. Thus, associations between genes and molecular, cellular, or benign phenotypes (e.g. increased and decreased enzyme activity, cell permeability, or pigmentation) were also excluded.

### Probability of drug approval analysis

Analysis of the odds of drug target-indication approval was conducted in R/3.5.2 following the methods of King et al.^30^. Since some OMIM disease genes were also associated with bidirectional phenotypic effects, we first replicated their analysis after removing those genes to estimate the odds of approval for drugs that target OMIM genes with unidirectional effects. We then repeated this analysis with our set of gene-trait associations with bidirectional effects replacing the OMIM genes in the original King et al. data set. We called the function annotate_target_indication_table_with_genetic_evidence() to match the set of target-indications with the best supporting genetic evidence. We then used the function replicate_table1() to estimate the relative risk of transition between different phases of clinical development using a range of MeSH term similarity thresholds (0–1 by increments of 0.01) to link drug indications with genetically associated diseases or traits. The maximum odds of transition from Phase I through approval occurred for indications with MeSH similarity >0.83.

### Drug approval sensitivity analyses

Sensitivity analyses were conducted by removing the evidence annotations for specific groups of BEST genes prior to calculating the relative risk of transition between clinical phases. The five largest phenotype categories among the identified BEST genes were lipids, clotting, reproduction, height, and glucose. Combined, these represented 45% of our targets. We repeated our analysis without the genetic evidence supporting these categories to see if the remaining phenotype categories would still show a significant signal for BEST genes. Our results indicate that the BEST genes from the remaining categories are still significantly enriched from approval with a slightly greater probability of transition from Phase I to Approval (RR = 4.15; 95% CI = [2.84, 5.45]; Supplementary Fig. 2).

Secondly, we performed an additional sensitivity analysis by excluding the genetic evidence supporting BEST genes from three areas previously described as having historically high clinical success rates (metabolic, endocrine, and hematological)^1^. Similarly, we show that even after removal of this evidence, there is still a significant increase in probability of success for the BEST genes in the remaining broad therapeutic categories (Supplementary Fig. 3).

Finally, we performed stratified analysis for the five phenotype categories with the largest number of BEST targets (hematic, cardiovascular, metabolic, endocrine, and developmental). These were the only categories that contained more than 5 BEST genes and thus were amenable to statistical analysis in isolation. We show that despite losing power by analyzing these categories independently, there is significant increase in probability of success within each category. Collectively these results demonstrate that our BEST selection criteria did not introduce a bias in one or more phenotypic categories that could explain the increased odds of approval we observed (Supplementary Fig. 4).

### Human growth databases

#### GWAS

We obtained summary statistics from the largest published meta-analysis for height based on imputed GWAS data from ~700,000 individuals^22^. In total, the 2067 non-repeating closest genes to genetic signals (within 1-MB window) were obtained (Supplementary Data 1 and 2, Methods section). We considered this the most comprehensive GWAS for height. Other approaches to map GWAS signals to genes such as co-localization using eQTL data were not suitable since they require gene expression data in the tissue most relevant to the phenotype under study. For height, this would be limb bone growth plates and there are currently no gene expression datasets available for this tissue^56,57^.

#### HGMD

We queried the “allmut” table from HGMD version v2019_2 looking for all pathogenic variants labeled as “DM” having either “short stature” and “tall stature or overgrowth” in the same genes (Supplementary Data 2).

#### OMIM

The list of OMIM genes related to growth disorders was previously described and was created using the keywords: short stature, overgrowth, skeletal dysplasia, and brachydactyly (Supplementary Data 2)^24^.

### Samples

#### UKBiobank

Data used for this research is available on the UK Biobank public repository. Data was accessed under application 41232. The demographics and patient characteristics for the 50,000 exome sequenced individuals has been previously described^58^. In short, individuals from the UK aged 45–75 were invited to participate. A baseline questionnaire and several measurements were taken. Electronic health records were also available. Standing height (field id 50) was measured in all individuals as part of the baseline assessment (Supplementary Table 1). We used GNU parallel to rapidly extract individual phenotype fields to CSV files^59^.

### Sequencing and variant calling

Sequencing protocol and details are described elsewhere^58^. In short, exomes were captured using a modified version of the IDT xGen Exome Research Panel 81 v1.0 and sequenced in pooled multiplexed samples using 75 base pair paired-end reads with two 10 base pair index reads on the Illumina NovaSeq 6000 platform using S2 flow cells. Reads were aligned to the GRCh38 genome reference with BWA-mem2. The WeCall variant caller was used to generate gVCF files (See URLs). A PVCF was created using the GLnexus joint genotyping tool^60^.

### Software

Genetic data analysis was performed on Hail 0.2.16 running on Apache Spark version 2.3.4.

### Exome sequencing data

We downloaded UK Biobank exome sequencing genotypes in Plink format for 49,960 individuals and 10,448,724 variants (“SBP” data) in May 2019.

We annotated and filtered individuals with data provided by UK Biobank. Specifically, we included individuals with the genetic ethnic grouping “Caucasian” (field 2206), no sex chromosome aneuploidies (field 22,019; effectively removing any classical Turner syndrome patients) and with a PCA-corrected heterozygosity between 0.17 and 0.21 (field 22019). After applying these filters, our dataset contained 41,190 individuals.

We removed related individuals by excluding samples meeting the criteria for relatedness and kinship set by UK Biobank (2nd degree relatives or closer) and that are listed in fields 22,011, 22,012, and 22,013. After applying this filter, our dataset contained 35,990 individuals.

We computed sample and variant QC metrics and applied additional filters that excluded sample with more than 80,000 non-reference variants, more than 200 singletons, a het/hom ration of <1.3 or >1.85, and transition/transversion ratio of >2.5, a call rate of <0.985 or a proportion of heterozygous sites in sequencing data that are also heterozygous in chip data of <0.98. We also excluded variants that had a call rate of <0.99 or a Hardy–Weinberg *p* < 1 × 10^−10^.

We annotated variants using the variant effect predictor (VEP)^61^ and extracted rare high impact variants. We defined those as variants with a minor allele frequency of less than 0.01 and whose impact as predicted by VEP is either high (protein truncation) or moderate (missense). We observed that a subset of those high-impact variants had an unexpectedly high allele frequency compared to the allele frequency of non-Finnish European (NFE) individuals in the genome aggregation database (gnomAD)^62^. We removed those variants if the observed allele count in our dataset was significantly different to what would be expected based on the NFE allele frequency in gnomAD (*p* < 1 × 10^−7^, binomial test). After applying those filters, our dataset contained 581,392 variants. The assignment of variants to genes was accomplished by using canonical VEP gene models.

### Quality control

After applying the filters described above, our exome sequencing dataset contained 34,284 individuals and 10,173,231 variants. There was no apparent clustering of the post-filtering European ancestry individuals by genotyping eigenvectors (Supplementary Figs. 25–26). The sample call rate was between 0.999941 and 1.0 (median: 0.999984) and the variant call rate was between 0.989909 and 1.0 (median: 1.0; Supplementary Figs. 27–28).

We observed between 37 and 111 (median: 68) rare high-impact variants per individual (Supplementary Fig. 29) and between 0 and 36 (median: 9) singletons per individual (Supplementary Fig. 30). Most individuals had a rare high-impact variant in less than 0.01 of genes (Supplementary Fig. 31).

### Gene set analyses

We constructed a list of 2,355 genes (Supplementary Data 5) that had evidence from GWAS and/or HGMD/OMIM. To assign a GWAS signal to a gene, we used the latest meta-analysis for height and took the list of 3290 predicted independent variants (Supplementary Data 1)^22^. Then we mapped the closest gene to each one of those lead variants and removed duplicates. That list of genes was compiled together with genes reported on HGMD and OMIM and genes were assigned one to five different annotations: GWAS, HGMD_SHORT, HGMD_TALL, OMIM-OVERGROWTH, and OMIM-SHORT (Supplementary Data 2).

We then queried this list of genes in the UKBiobank exome dataset. Here 1970 genes had at least 20 carriers (combined rare missense and LoF) and were considered for the analyses. Genes had a mean of 204 rare protein coding variants (Supplementary Fig. 32).

### Statistical association analyses

Standing height measurements were first split by gender and then normalized to standard deviations adjusted for age and first five principal components using a linear regression implemented on the R package. Linear and logistic regression analyses for single variants were computed using Hail 0.2. Effect estimates (beta) were calculated from linear and logistic regression for quantitative and discrete analyses respectively.

We grouped rare missense and loss-of-function variation per gene using GENCODE canonical transcripts. We applied SKAT and burden tests on missense variants and predicted protein truncating variants (PTVs) with a minor allele frequency <0.0001 using Hail 0.2.

For the association analysis on gene-sets and ISS we grouped genes in the following way:

Bidirectional: Genes present in HGMD_SHORT, HGMD_TALL, OMIM-OVERGROWTH, OMIM-SHORT. Tall: Genes present in HGMD_TALL or OMIM-OVERGROWTH but not in HGMD_SHORT or OMIM _SHORT; Short: Genes present in HGMD_SHORT and OMIM _SHORT but not in HGMD_TALL or OMIM-OVERGROWTH.

### SNP chip data and polygenic scores

We used summary statistics from a large GWAS of human height to compute a height polygenic score (PS) for UK Biobank participants^24^. Specifically, we downloaded UK Biobank imputed SNP chip genotypes in BGEN format for 487,409 individuals. We exported genotypes for 2,540,786 variants for which GWAS summary statistics were available to Plink format. We then used LDpred^63^ version 1.0.7 to compute a height PS for 49,796 individuals for which exome sequencing data was available. For the coordination step of LDpred we used a linkage disequilibrium reference panel of 2000 randomly selected UK Biobank participants of European ancestry for which exome data was not available. For variant filtering, generating SNP weights and PS scoring we used default LDpred parameters. Linear regression analyses were used to estimate the effect of PS as a categorical variable (1–5) on height. Logistic regression analyses were used to estimate the effect of PS as a categorical variable (1–5) on ISS, groups with low number of ISS cases (standard error >100) were removed.

### Selection of NPR2 variants for functional assays

A total of 39 NPR2 protein coding variants were selected to investigate their functional effect at a cellular level (Supplementary Table 4). Fourteen of those variants had been previously described in the literature and are not explicitly found in our study. Seven variants were found in our exome dataset in single individuals with height >1 SD above the mean and were labeled as potential gain of function. Nine variants were found in individuals with height <2 SD below the mean and were labeled as potential loss of function. Three variants with neutral effect on height were tested. Four variants with MAF > 0.001 (p.A164G, p.R601H, p.E609K, and p.R358W) were added to complement the allelic series.

### Transfection functional assays and assay for cGMP analyses

HEK293T cells were cultured in Dulbecco’s modified Eagle’s medium (DMEM) supplemented with 10% fetal bovine serum, penicillin/streptomycin and Glutamax (Gibco) at 37 °C with 0.05 CO2. Transfection was performed in triplicate using a 96-well Shuttle nucleofector (Lonza). Briefly, 250 ng of plasmid per 2 × 10^5^ cells was transfected using Amaxa solution SF (Lonza) and program CA-138. Three days after transfection, cells were then incubated in DMEM containing 0.75 mM IBMX (3-isobutyl-1-methylxanthine; Enzo life sciences) for 15 min. The cells were next treated with 6.67 nM of BMN 111 (CNP)^52^ and incubated for another 15 min. The reaction was terminated with 40 μl of lysis solution, and the cGMP concentration was measured by a competitive enzyme immunoassay (Molecular Devices, R8075). Luciferase levels were measured using the ONE-Glo Assay (Promega) and the results are presented as the cGMP/luciferase normalized to the wild-type plasmid transfected cells. For the dose-response curves, the CNP concentrations assayed in triplicate were 33.33, 6.67, 1.33, 0.27, 0.053, 0.0107, and 0.002133 nM. The cGMP concentrations were assayed as above and cellular concentrations calculated assuming 1.6E5 cells/well with a diameter of 35 μm.

### Polygenic Scores and NPR2 functional data modeling

For all individuals carrying a rare variant in *NPR2*, for which functional assay data was available, we fit a generalized additive model to predict measured height (Z-transformed) as a function of their PS for height (Z-transformed) and the percent of wild-type cGMP observed for their *NPR2* variant (log base 2 transformed). Models were fit using the gam() function from package mgcv v1.8-31 in R version 3.6.1. A gamma parameter of 0.3 was chosen to minimize the amount of smoothing. Values smaller than this resulted in unstable models that produced unrealistic height predictions.

### CRISPR/Cas9 knockout of *NPR2* in rat chondrosarcoma chondrocytes

The swarm rat chondrosarcoma (RCS) cell line^64^ was cultured with DMEM containing 10% fetal bovine serum, penicillin/streptomycin, and Glutamax (Gibco) at 37 °C with 0.05 CO2. RCS cells were harvested with TrypLE Express Enzyme (Life Technologies) and transfected with RNPs formed with guide RNA containing ATTO 550 labeled tracrRNA (IDT 1075928) and crRNA targeting NPR2, with PAM in bold:

gRNA1 5′- CCCACTACTTCACCATCGAG**GGG**-3′

gRNA2 5′- CATTACACGGGCACTTCAAT**TGG**-3′

Briefly, equimolar ratios of crRNA and tracrRNA were annealed and 12 pmol was complexed with 104 pmol HiFi Cas9 (IDT 1081061) according to the manufacturer’s protocol. 2E5 cells were transfected with 500 ng of GFP mRNA (TriLink Biotechnologies, cat# L-7201) or RNP using a 96-well Shuttle nucleofector (Lonza) with Amaxa solution SF (Lonza) and program CA-150. Post transfection, each well was split and seeded into two wells of a 96-well plate. Three days after transfection, single cells were sorted into 96-well plates containing 200 μl of media using a FACSMelody cell sorter (BD Biosciences); the plates were monitored biweekly by imaging (Cell Metric, Solentim) when confluent cells were expanded into six-well plates and genotyped. Genomic DNA was extracted using Quanta Extracta solution (Quanta Biosciences, cat #95091). DNA amplicons covering the gRNA target region were amplified and analyzed by PacBio sequencing following the manufacturer’s instructions (Pacific Biosciences, 101-791-800 Version 01). Amplicon libraries were prepared using primers with universal sequences and the locus specific primers FW 5′-CATCCCTGCTACTGGTGGTG-3′ and REV 5′-CTCCACCACCAAACCTGAACT-3′. Sequencing was performed using the Sequel System using the SMRT Cell 1 M v3 and acquiring 10 h movies. Demultiplexing and circular consensus sequence analysis and FASTQ generation was performed using the SMRTlink 6.0 software (Pacific Biosciences). To analyze amplicon sequencing, Geneious Prime 2019.0.4 software was used for quality assessment, quality filtering, reference alignment, and calculating indels (Supplementary Note).

### Power analysis

We used the PAGEANT^65^ method to estimate the power of the current study using the SKAT method on the following two scenarios: (a) our study design involved an **α** = 0.002 (adjusting for 25 gene set tests), we considered a variance explained of 0.0009. (b) A study would require 400,000 samples to identify single genes at *α* = 2.5 × 10^−6^, a maximum of 1000 causal genes, and considering 0.05 of variance explained for protein-altering variants genome-wide.

### Reporting summary

Further information on research design is available in the Nature Research Reporting Summary linked to this article.

## Supplementary information

Supplementary Information

Dataset 1

Dataset 2

Dataset 3

Dataset 4

Dataset 5

Dataset 6

Dataset 7

Reporting Summary

Description of Additional Supplementary Files

## Data Availability

Data used for this research is available on their respective public repositories. The UK Biobank exome sequence data was obtained under application 41,232. These data are available to approved researchers through the UKB Data Showcase at http://ukbiobank.ac.uk/ Full table of genetic evidence and approved drugs is available at: https://github.com/AbbVie-ComputationalGenomics/genetic-evidence-approval. The Human Gene Mutation Database can be obtained with a license from https://digitalinsights.qiagen.com The OMIM database can be found at https://www.omim.org/

## Code availability

Code used for this research is available upon request
