# Supplementary material for: Identifying therapeutic drug targets using bidirectional effect genes
Source: Nat Commun. 2021 Apr 13;12:2224. doi: 10.1038/s41467-021-21843-8 (PMC8044152; doi:10.1038/s41467-021-21843-8)
Supplement: Supplementary file 1 — Supplementary Information [file 41467_2021_21843_MOESM1_ESM.pdf]

Supplementary Information

Identifying therapeutic drug targets using bidirectional effect genes

Karol Estrada*[1], Steven Froelich[1], Arthur Wuster[1], Christopher R. Bauer[1,] Teague Sterling[1], Wyatt T. Clark[1], Yuanbin Ru[1], Marena Trinidad[1], Hong Phuc Nguyen[1], Amanda R. Luu[1], Daniel J. Wendt[1], Gouri Yogalingam[1], Guoying Karen Yu[1], Jonathan H. LeBowitz[1], Lon R. Cardon[1]

[1]BioMarin Pharmaceutical Inc., Novato, CA 94949, USA

*Corresponding author
Email: karol.estrada@bmrn.com

Table of Contents

Supplementary Figure 1. Estimated odds ratio for transition from Phase I to Approved Clinical Trials 3
Supplementary Figure 2. Estimated odds ratio for transitions between various clinical trial phases for BEST genes excluding targets in the in five disease categories (i.e., Lipids, clotting, reproduction, height and glucose) 3
Supplementary Figure 3. Estimated odds ratio for transitions between various clinical trial phases for BEST genes excluding targets in the in three disease categories (i.e., metabolic, endocrine, hematological) 4
Supplementary Figure 4. Estimated odds ratio for Phase I to Approval transition for BEST genes stratified by disease category .. 4
Supplementary Figure 5. Flow chart describing the steps followed to validate bidirectional genes for height. 5
Supplementary Figure 6. Groups of genes analyzed to identify five genes that regulate height. 6
Supplementary Figure 7. Regional plot near the IGF1R gene. 7
Supplementary Figure 8. Regional plot near the FGFR3 gene 8
Supplementary Figure 9. Regional plot near the NPPC gene 9
Supplementary Figure 10. Regional plot near the NPR2 gene. 10
Supplementary Figure 11. Odds Ratio for Idiopathic Short Stature (ISS) on highlighted gene-sets. 11
Supplementary Figure 12. Effect of FGFR3 missense and LoF variants on height stratified by PRS. 12
Supplementary Figure 13. Effect of IGF1R missense and LoF variants on height stratified by PRS. 13
Supplementary Figure 14. Effect of NPPC missense and LoF variants on height stratified by PRS. 14
Supplementary Figure 15. Effect of NPR2 missense and LoF variants on height stratified by PRS. 15
Supplementary Figure 16. Effect of SHOX missense and LoF variants on height stratified by PRS. 16
Supplementary Figure 17. Mean effect on height of PTV carriers standardized by PRS quintiles 17
Supplementary Figure 18. Mean effect on height of missense carriers standardized by PRS quintiles 18
Supplementary Figure 19. Effect of having a protein altering variant in five genes using non-carriers at PRS 1 quintile as reference 19
Supplementary Figure 20. Effect of having a protein altering variant in five genes using non-carriers at PRS 2 quintile as reference 20
Supplementary Figure 21. Effect of having a protein altering variant in five genes using non-carriers at PRS 3 quintile as reference 21
Supplementary Figure 22. Effect of having a protein altering variant in five genes using non-carriers at PRS 4 quintile as reference 22
Supplementary Figure 23. Effect of having a protein altering variant in five genes using non-carriers at PRS 5 quintile as reference 23
Supplementary Figure 24. Plot of PRS vs Height in all exome sequenced individuals. 24
Supplementary Figure 25. Eigenvectors 1 and 2 for 34,284 individuals with exome sequencing passing filters. 25
Supplementary Figure 26. Eigenvectors 1 and 3 for 34,284 individuals with exome sequencing passing filters. 25
Supplementary Figure 27. Call rate distribution for samples 26
Supplementary Figure 28. Call rate distribution for variants. 26
Supplementary Figure 29. Rare high-impact variants per individual 27
Supplementary Figure 30. Rare high-impact singletons per individual 27
Supplementary Figure 31. Proportion of individuals (out of 34,284) with a rare high-impact variant for each gene (n=19,117) .. 28
Supplementary Figure 32. Distribution of minor allele counts in the 1918 genes included in gene set 29

Supplementary Note. 30

Supplementary Tables.

Supplementary Table 1. Study descriptive summaries for UK Biobank samples 32
Supplementary Table 2. SKAT Association results for the 19 gene sets tested on height 33
Supplementary Table 3. Proposed mechanisms of identified genes for growth regulation 34
Supplementary Table 4. NPR2 mutations tested in functional experiments 35
Supplementary Table 5. Median height of PRS x cGMP category in NPR2 carriers 36

Supplementary Figures

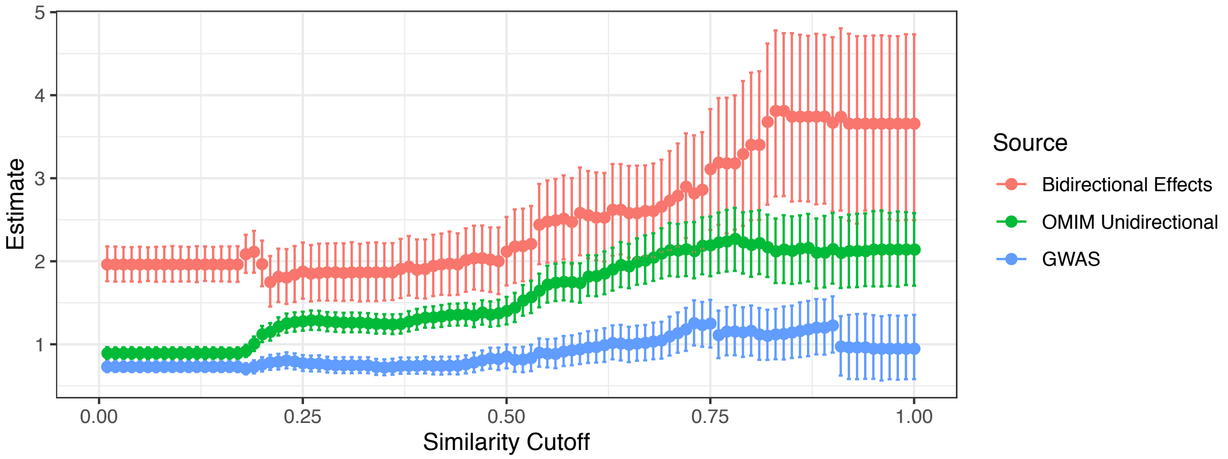

Supplementary Figure 1. Estimated odds ratio for transition from Phase I to Approved Clinical Trials

Measure of center for the error bars represent the estimated odds ratios and the error bars represent the 95% confidence intervals. Bidirectional effect supported data is shown in red, OMIM unidirectional support is shown in green, and GWAS support is shown in blue. n=26,884 target – indication pairs.

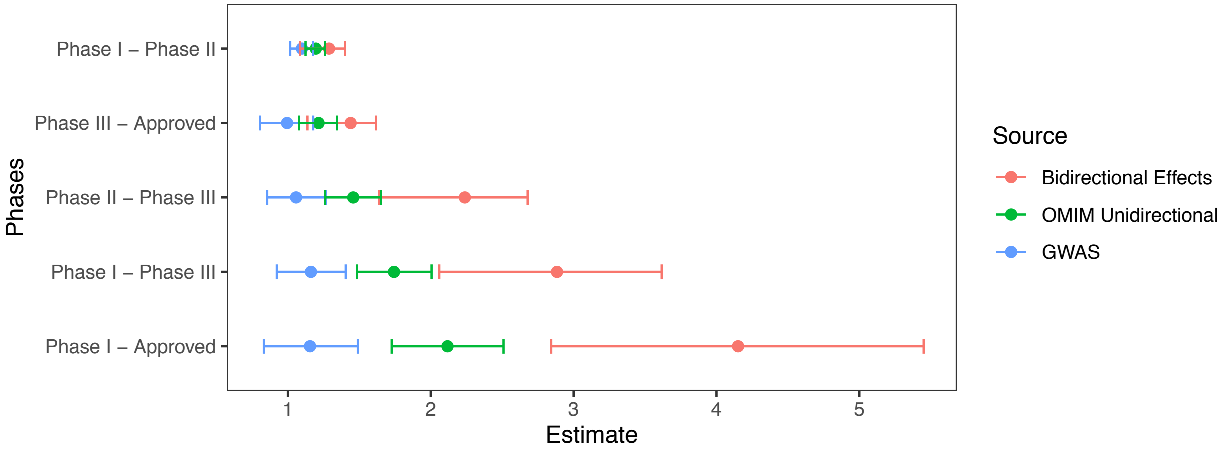

Supplementary Figure 2. Estimated odds ratio for transitions between various clinical trial phases for BEST genes excluding targets in the in five disease categories (i.e., Lipids, clotting, reproduction, height and glucose)

Measure of center for the error bars represent the estimated odds ratios and the error bars represent the 95% confidence intervals. Bidirectional effect supported data is shown in red, OMIM unidirectional support is shown in green, and GWAS support is shown in blue. n=26,884 target – indication pairs.

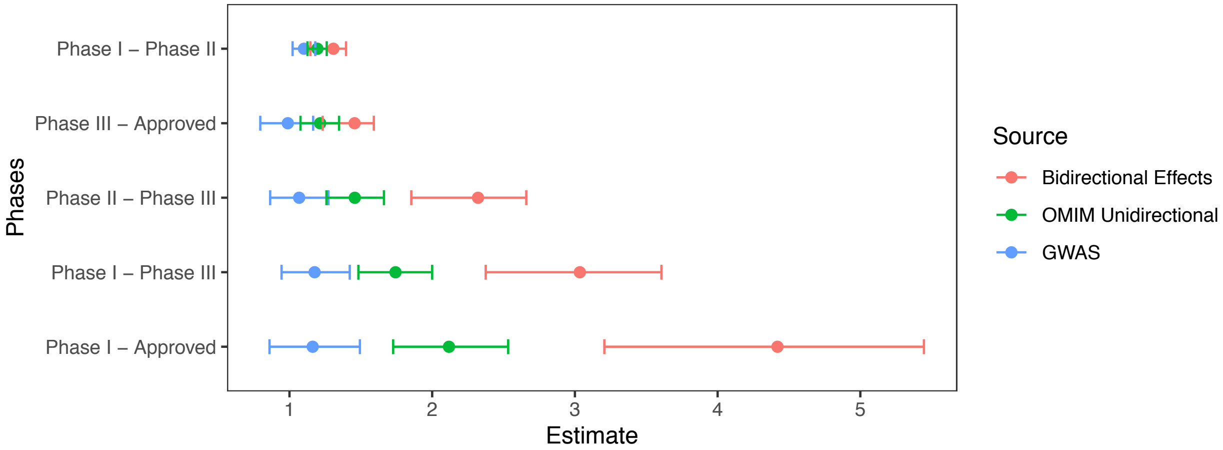

Supplementary Figure 3. Estimated odds ratio for transitions between various clinical trial phases for BEST genes excluding targets in the in three disease categories (i.e., metabolic, endocrine, hematological).

Measure of center for the error bars represent the estimated odds ratios and the error bars represent the 95% confidence intervals. n=26,884 target – indication pairs.

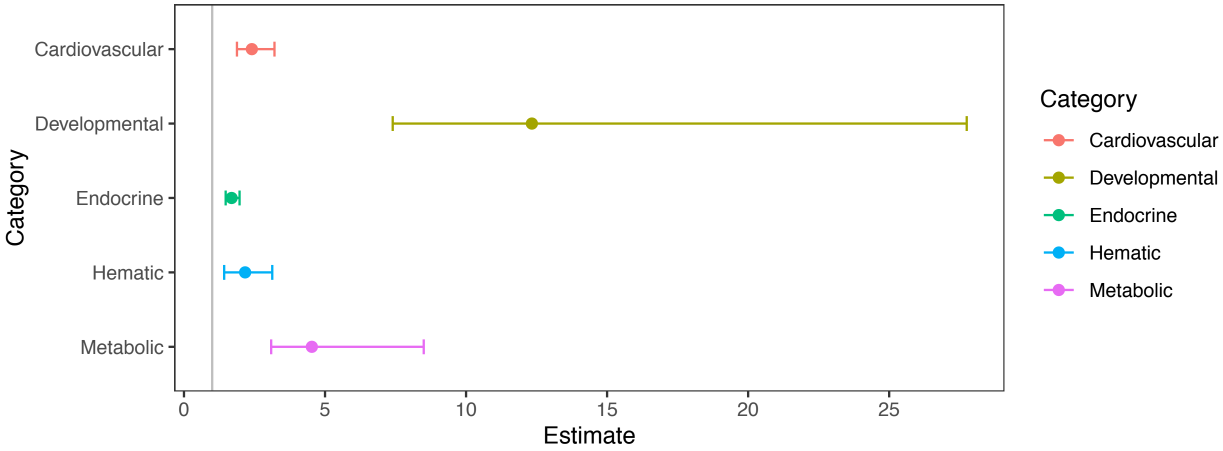

Supplementary Figure 4. Estimated odds ratio for Phase I to Approval transition for BEST genes stratified by disease category.

Measure of center for the error bars represent the estimated odds ratios and the error bars represent the 95% confidence intervals. n=26,884 target – indication pairs.

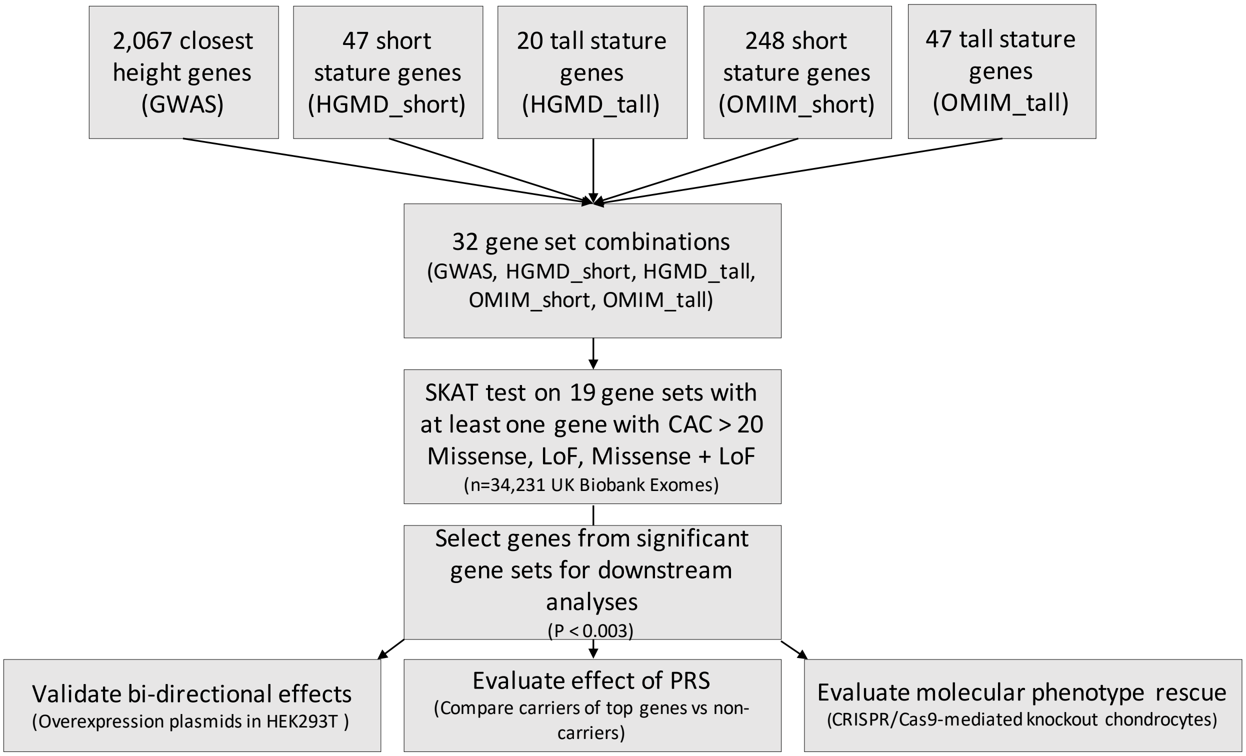

Supplementary Figure 5. Flow chart describing the steps followed to validate bidirectional genes for height.

PRS, Polygenic Risk Score computed using previous meta-analysis of common variants. GWAS, Genome-Wide Association Study; HGMD, Human Gene Mutation Database; OMIM, Online Mendelian Inheritance in Man; SKAT SNP-set (Sequence) Kernel Association Test

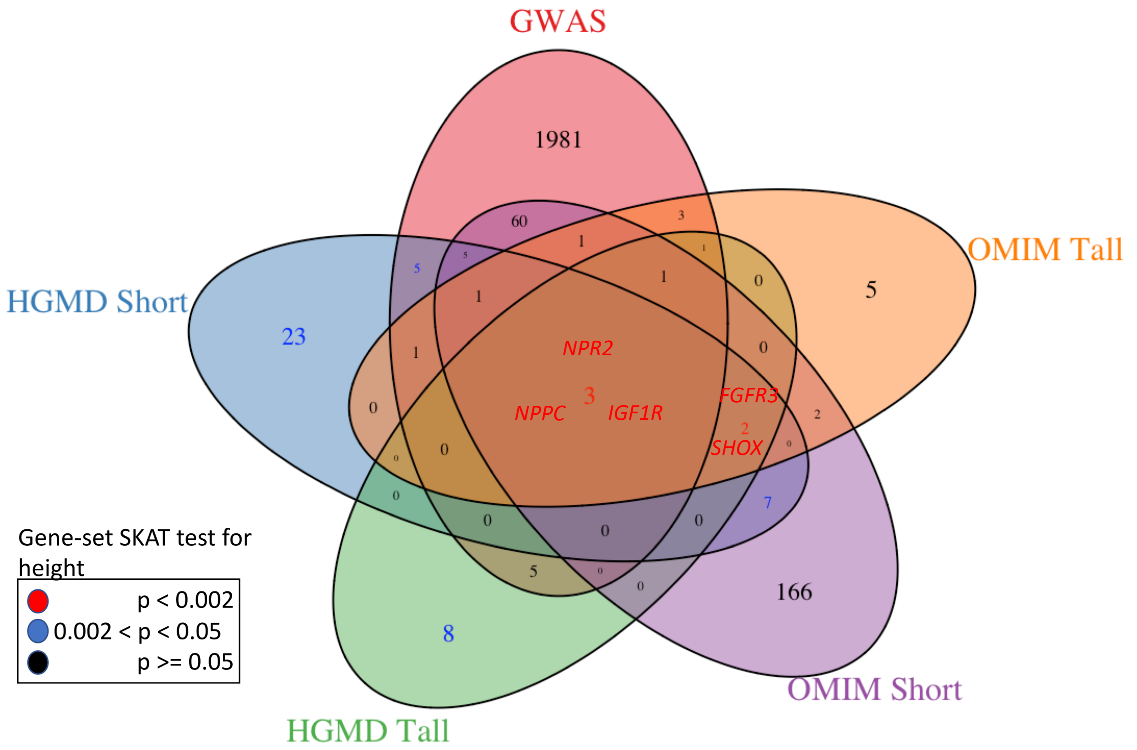

Supplementary Figure 6. Groups of genes analyzed to identify five genes that regulate height.

The color codes of the gene names or the number of genes in the gene set corresponds to the SKAT test p-value for the association with height on 34,284 UK Biobank exome sequences. P-values calculated with a 2-sided test without adjustment for multiple testing.

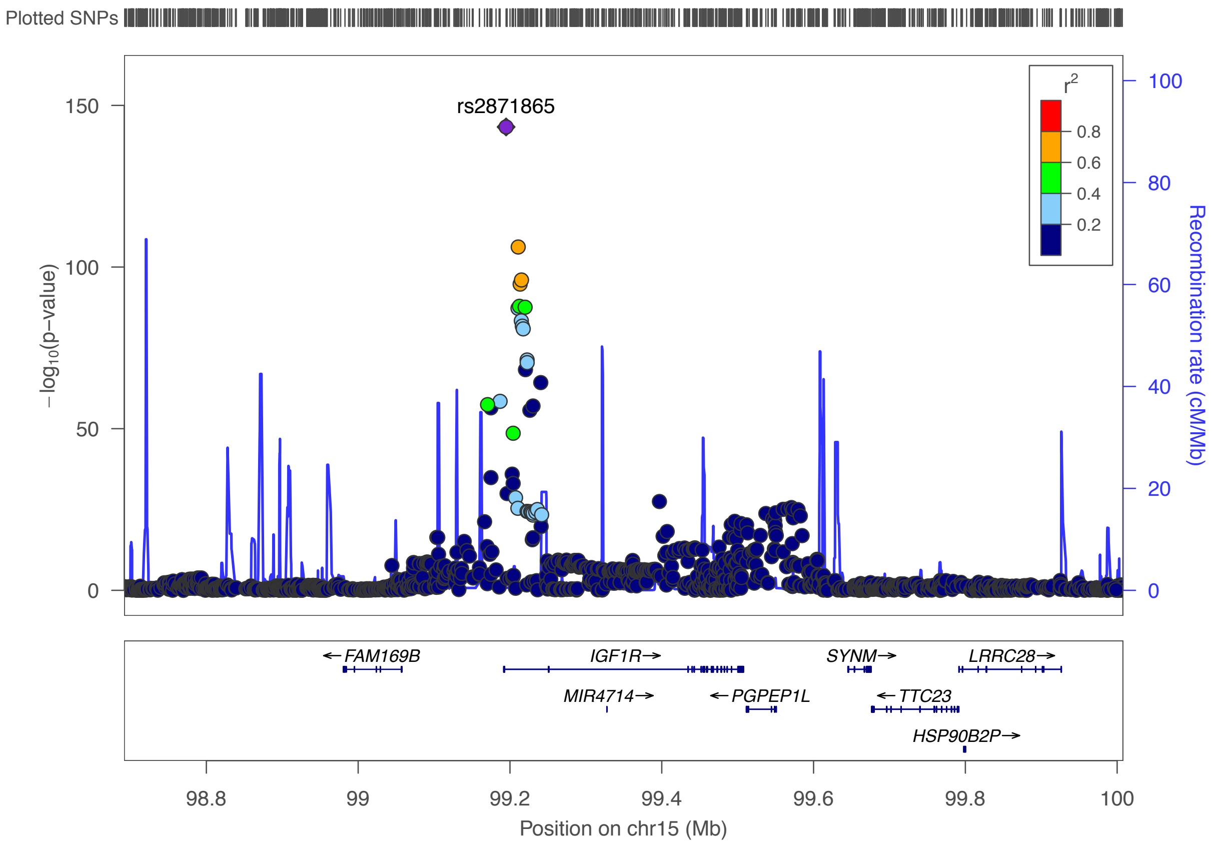

Supplementary Figure 7. Regional plot near the IGF1R gene.

The Y-axis represents the -log10 p-value for height in the Yengo et al. meta-analysis. P-values calculated from linear regression analysis using a 2-sided test without adjustment for multiple testing.

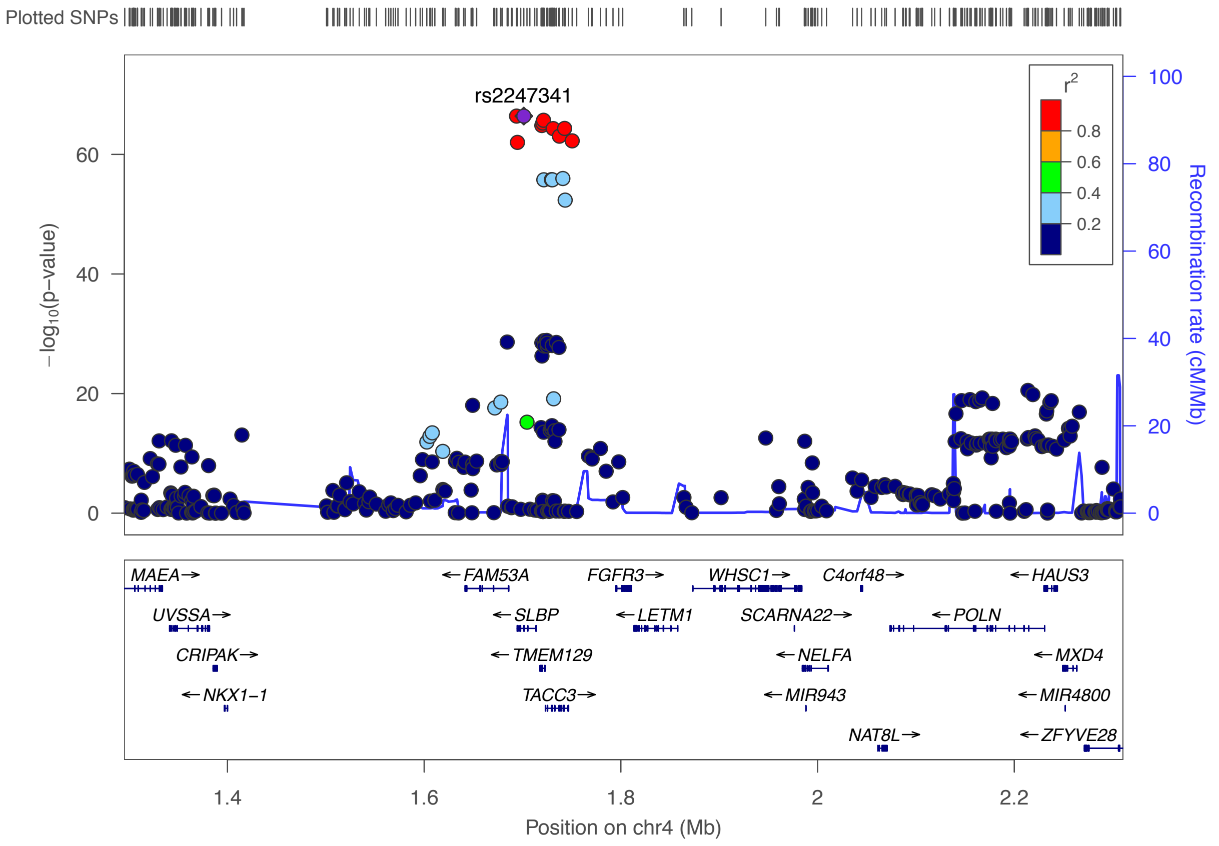

Supplementary Figure 8. Regional plot near the FGFR3 gene

Y-axis represents the -log10 p-value for height in the Yengo et al. meta-analysis. P-values calculated from linear regression analysis using a 2-sided test without adjustment for multiple testing.

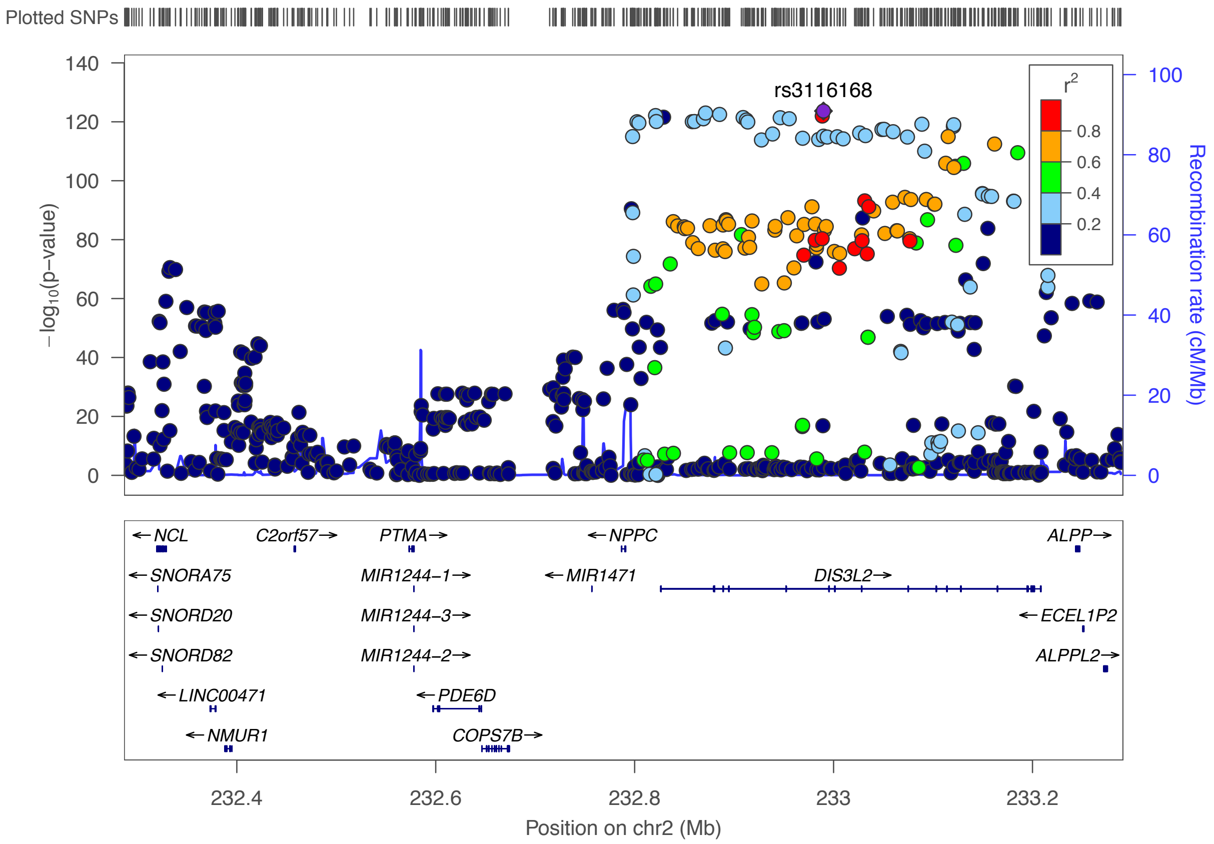

Supplementary Figure 9. Regional plot near the NPPC gene

Y-axis represents the -log10 p-value for height in the Yengo et al. meta-analysis. P-values calculated from linear regression analysis using a 2-sided test without adjustment for multiple testing.

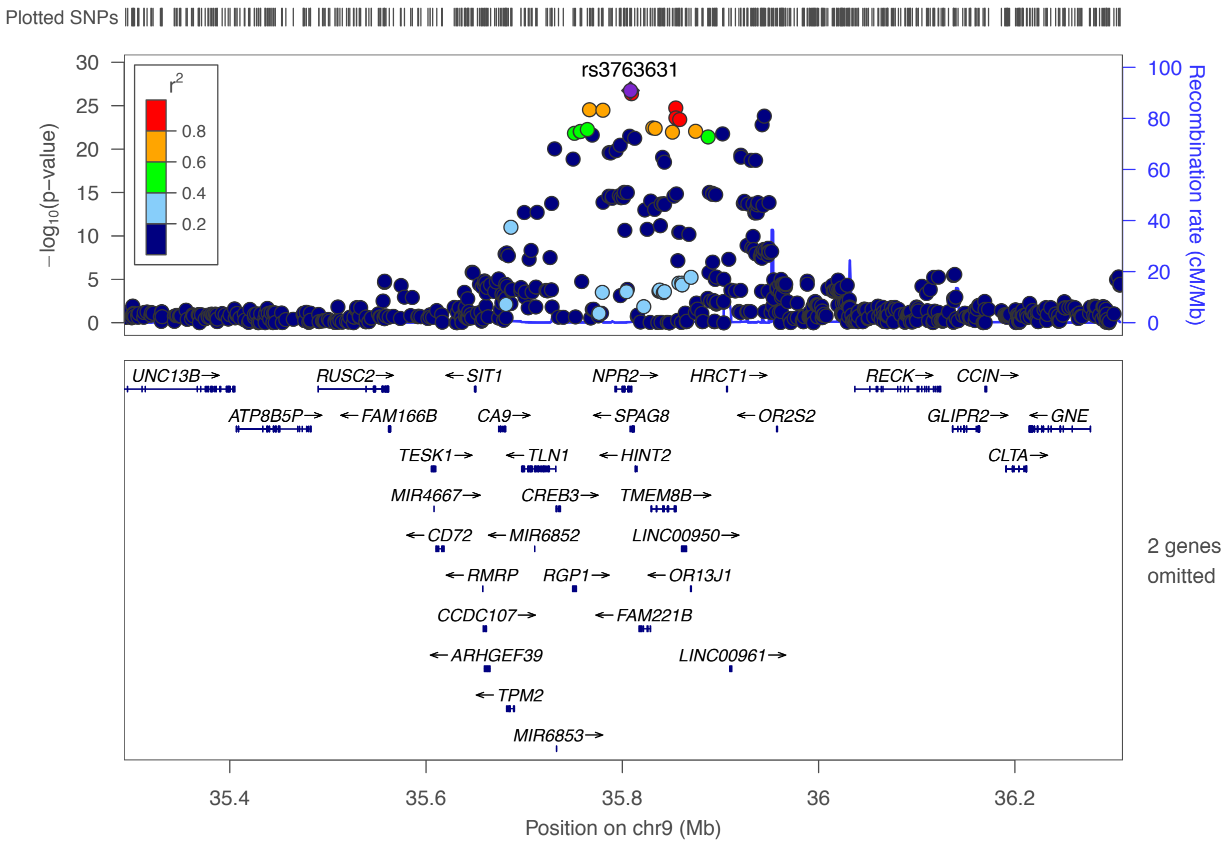

Supplementary Figure 10. Regional plot near the NPR2 gene.

Y-axis represents the -log10 p-value for height in the Yengo et al. meta-analysis. P-values calculated from linear regression analysis using a 2-sided test without adjustment for multiple testing.

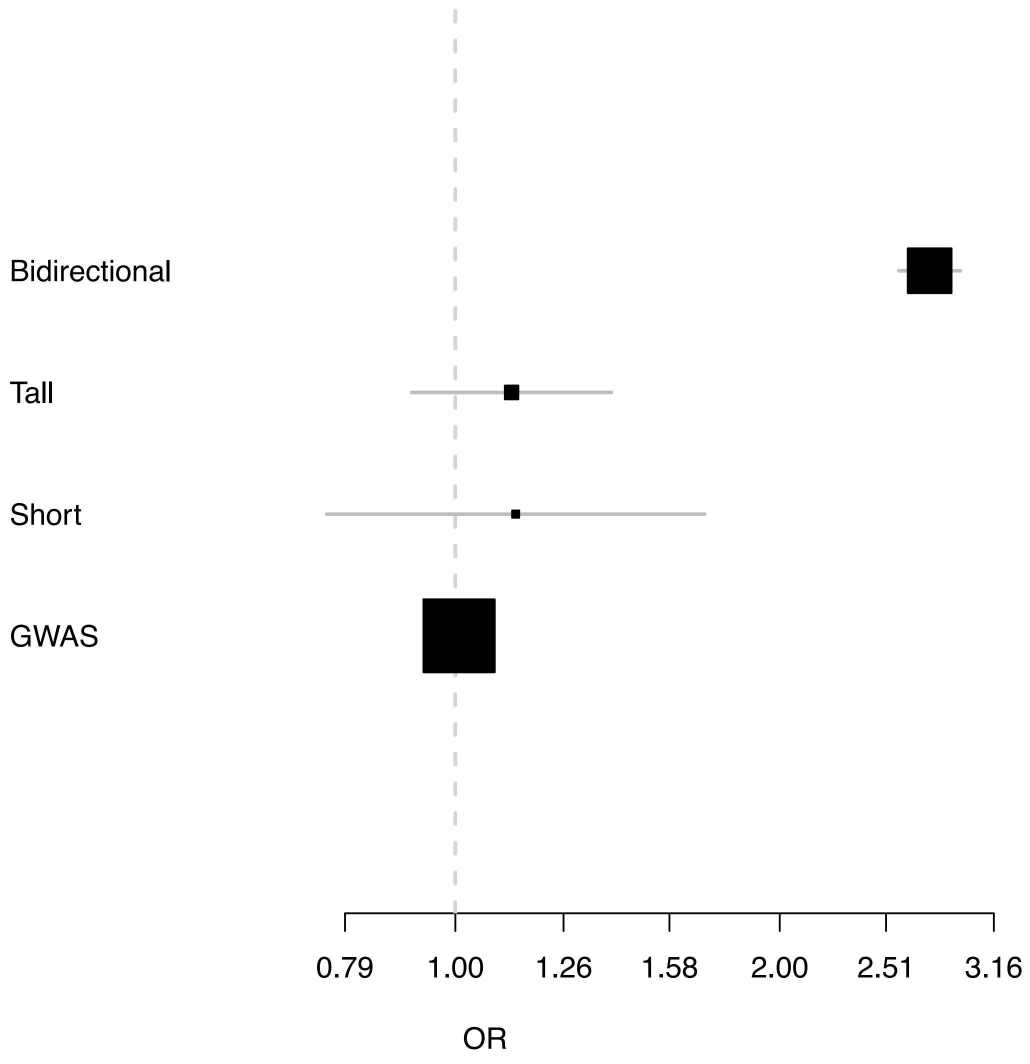

Supplementary Figure 11. Odds Ratio for Idiopathic Short Stature (ISS) on highlighted gene-sets.

Center in the error bar represent the estimate and error bars represent 95% CI. Bidirectional n=5 genes; Tall n=20 genes; Short n=47 genes; GWAS n=2,067 genes

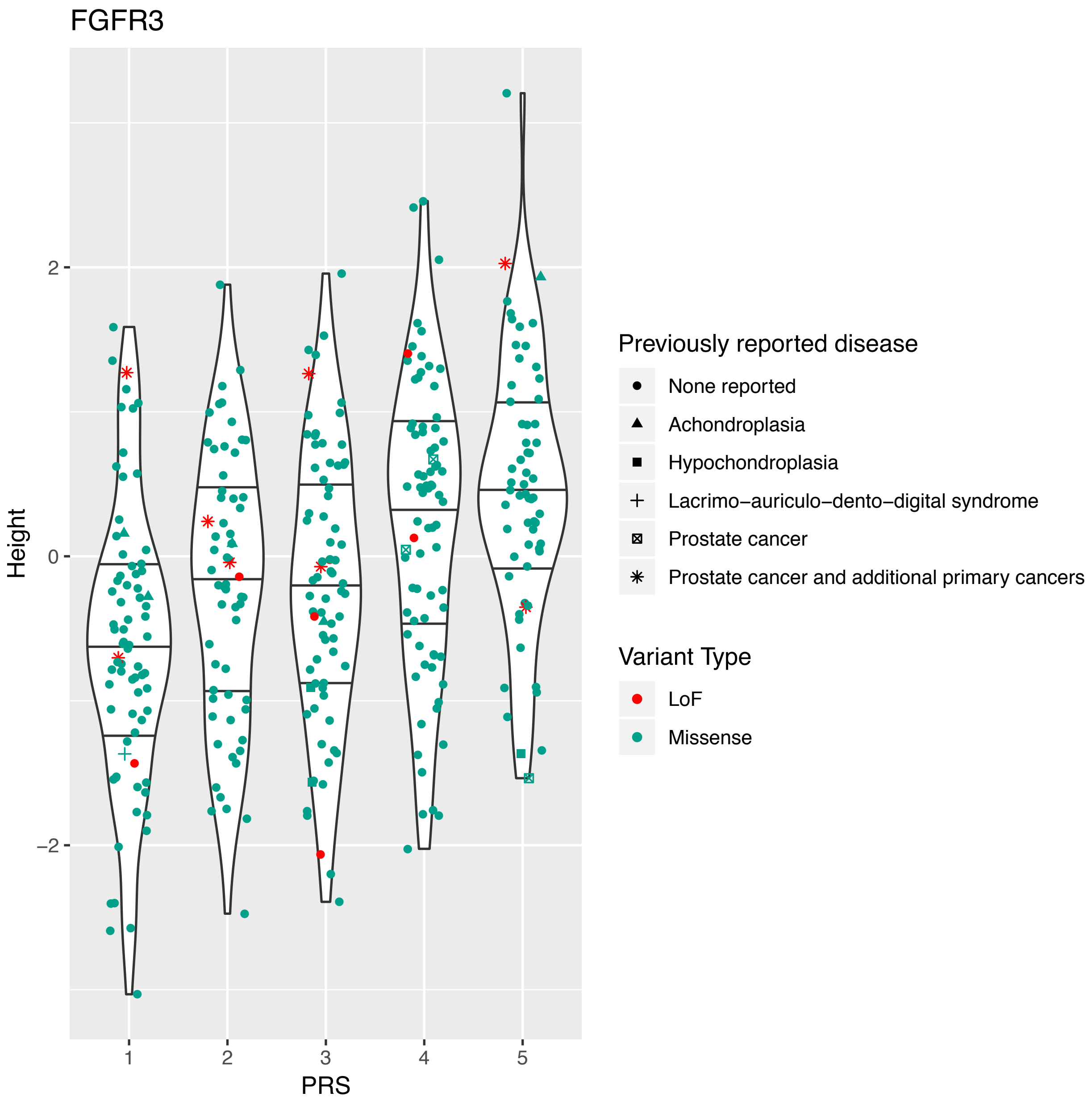

Supplementary Figure 12. Effect of FGFR3 missense and LoF variants on height stratified by PRS

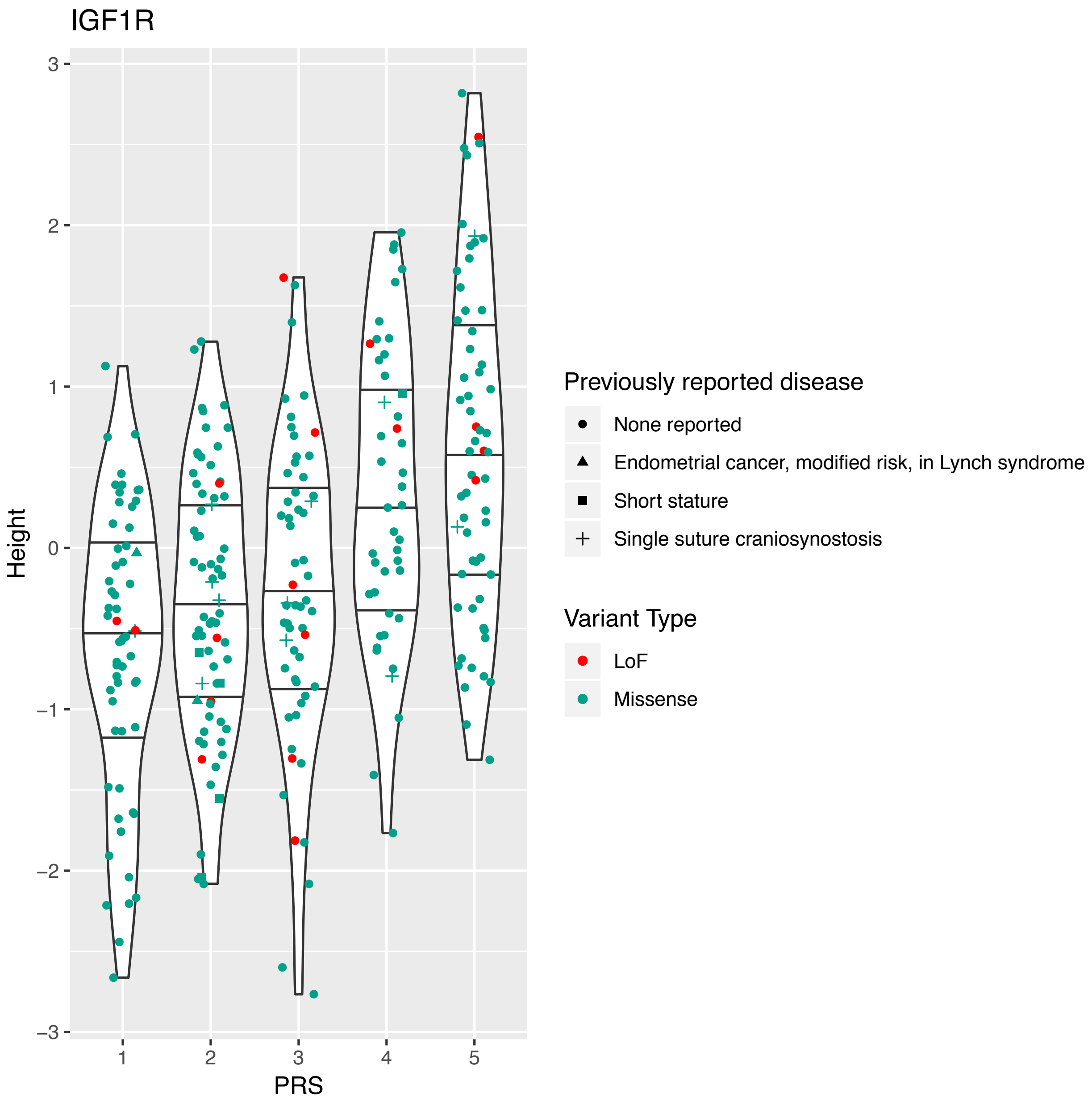

Supplementary Figure 13. Effect of IGF1R missense and LoF variants on height stratified by PRS.

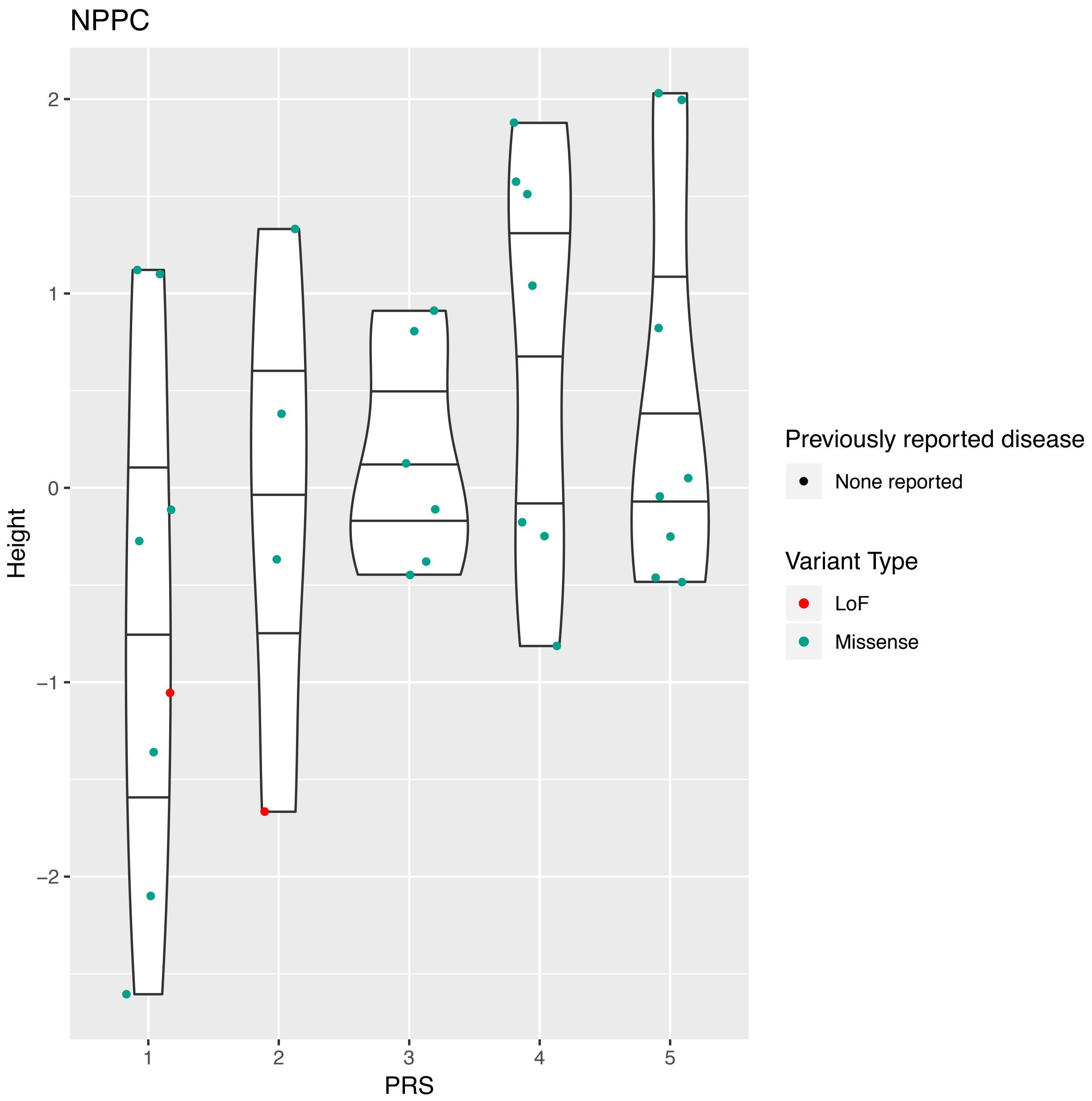

Supplementary Figure 14. Effect of NPPC missense and LoF variants on height stratified by PRS.

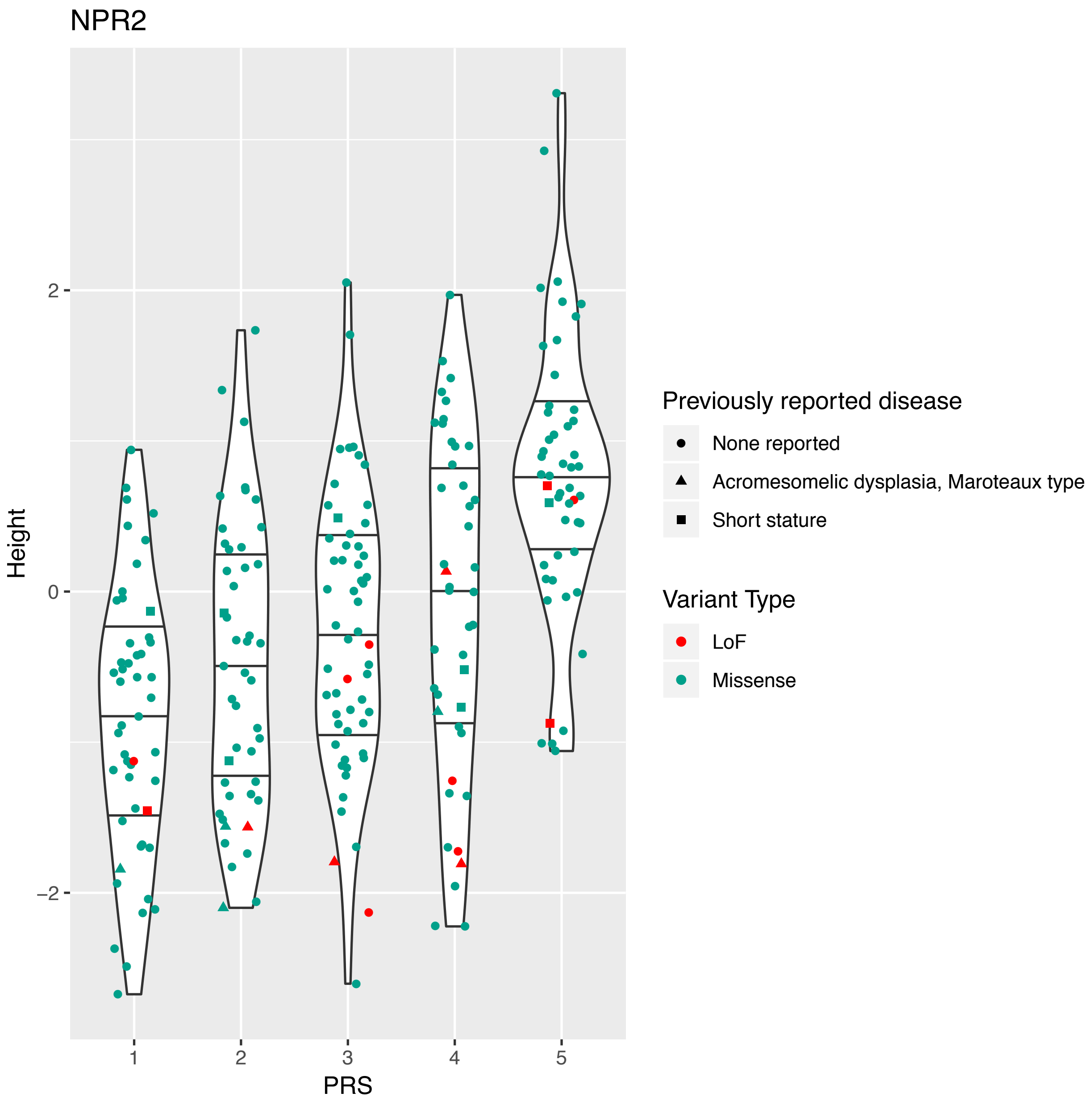

Supplementary Figure 15. Effect of NPR2 missense and LoF variants on height stratified by PRS.

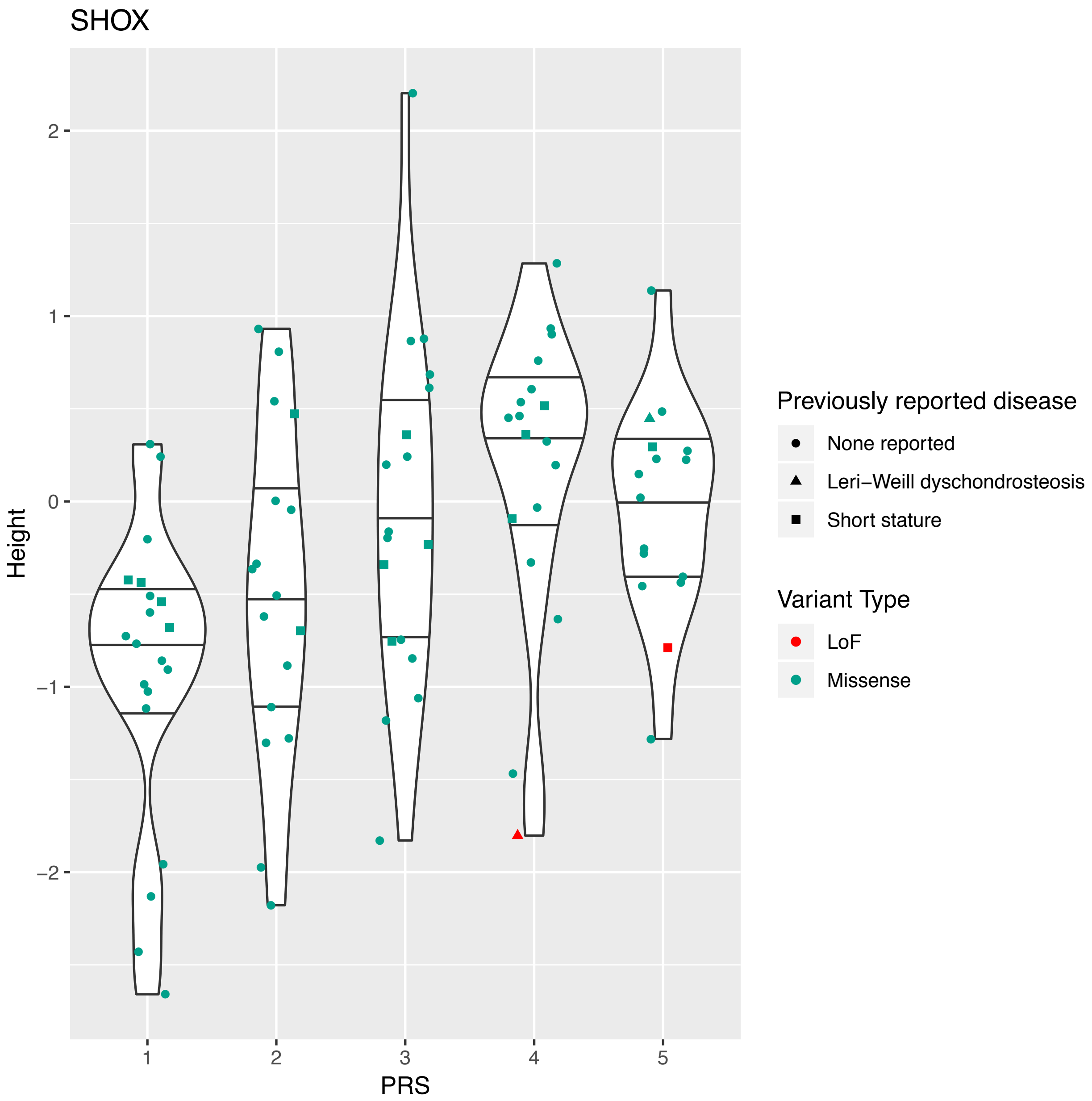

Supplementary Figure 16. Effect of SHOX missense and LoF variants on height stratified by PRS.

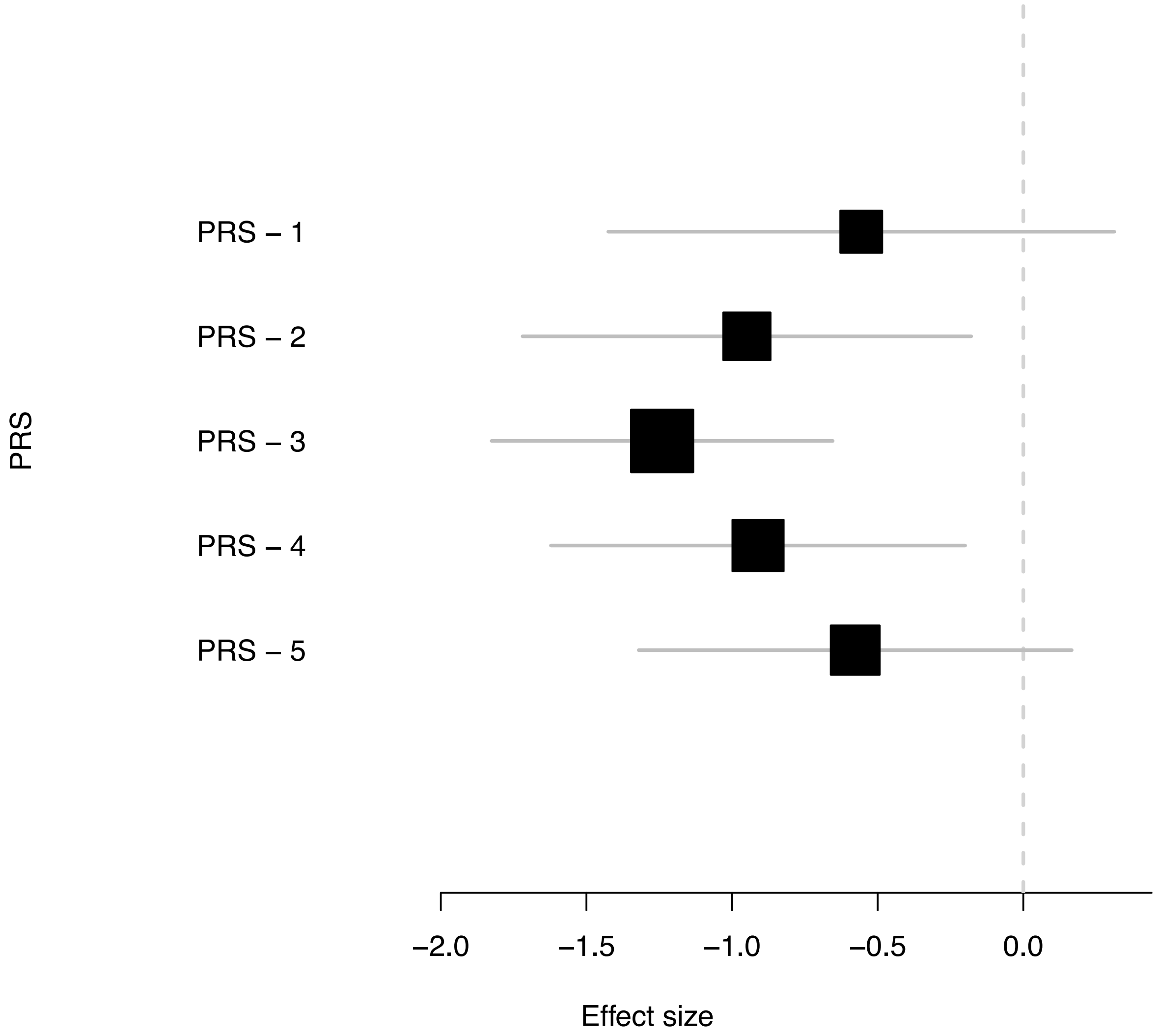

Supplementary Figure 17. Mean effect on height of PTV carriers standardized by PRS quintiles.

Measure of center for the error bars represent the mean effect. Error bars represent 95% confidence intervals. n=30 samples

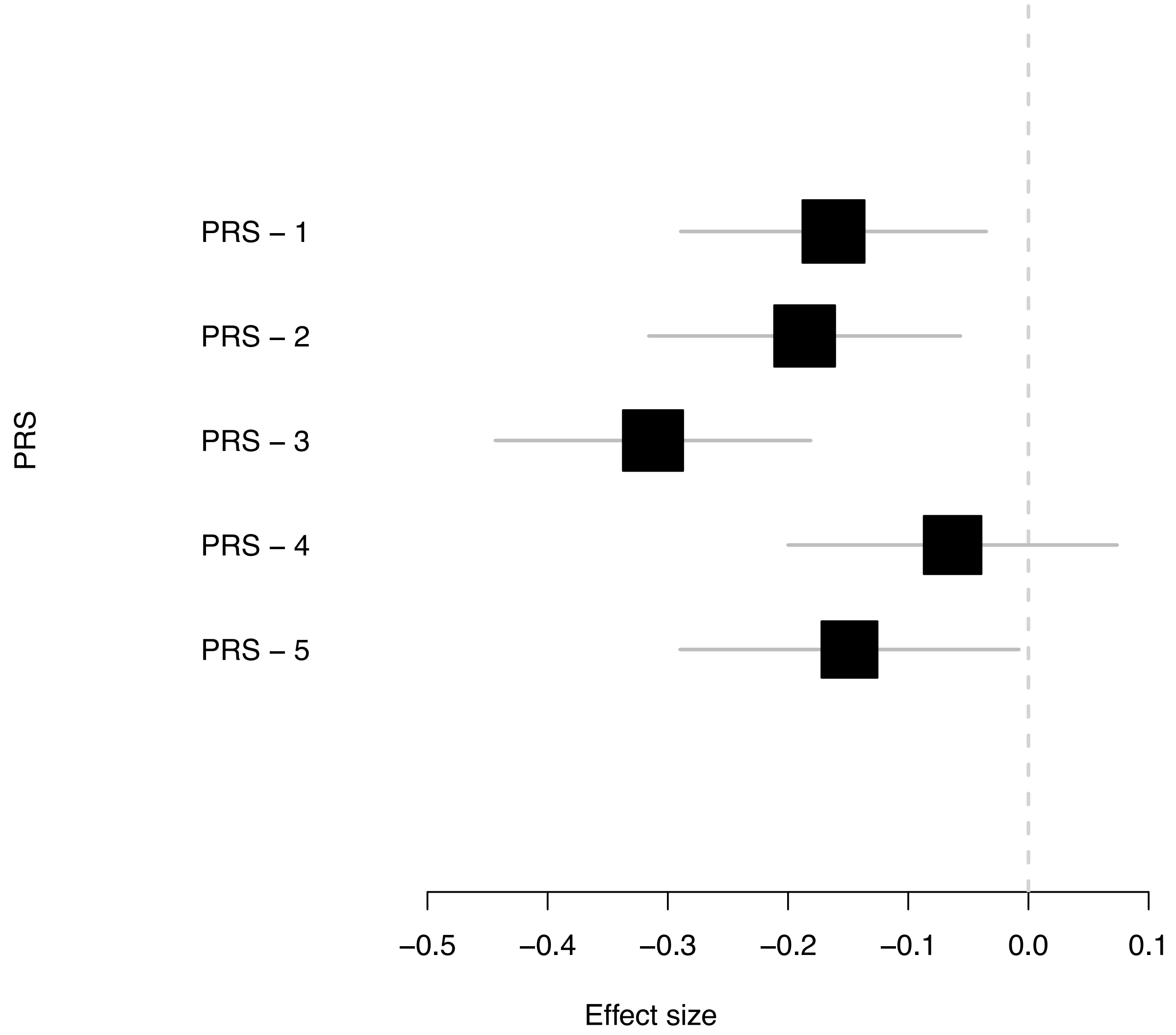

Supplementary Figure 18. Mean effect on height of missense carriers standardized by PRS quintiles.

Measure of center for the error bars represent the mean effect. Error bars represent 95% confidence intervals. n=907 samples

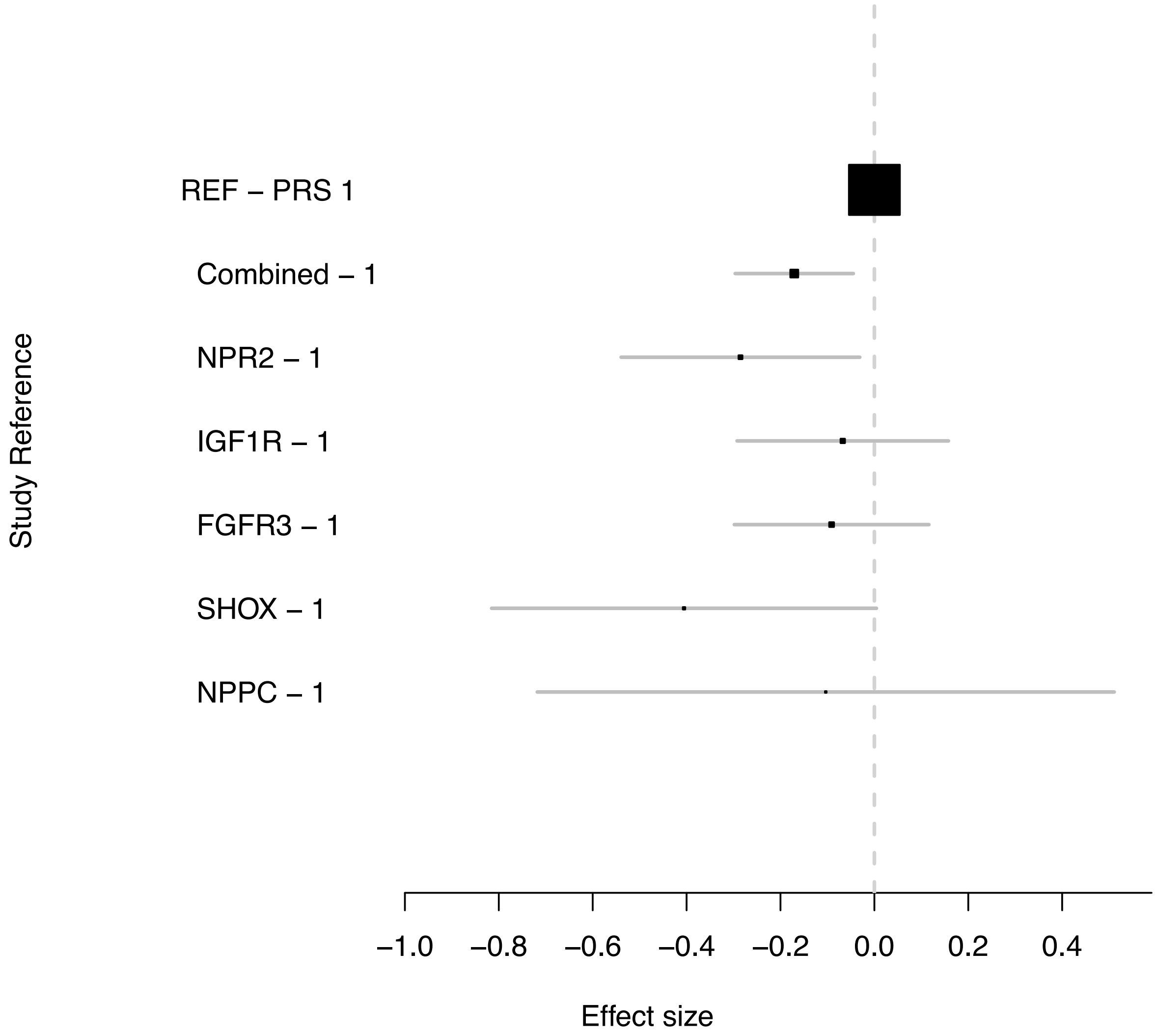

Supplementary Figure 19. Effect of having a protein altering variant in five genes using non-carriers at PRS 1 quintile as reference.

Measure of center for the error bars represent the mean effect. Error bars represent 95% confidence intervals. n=937 samples

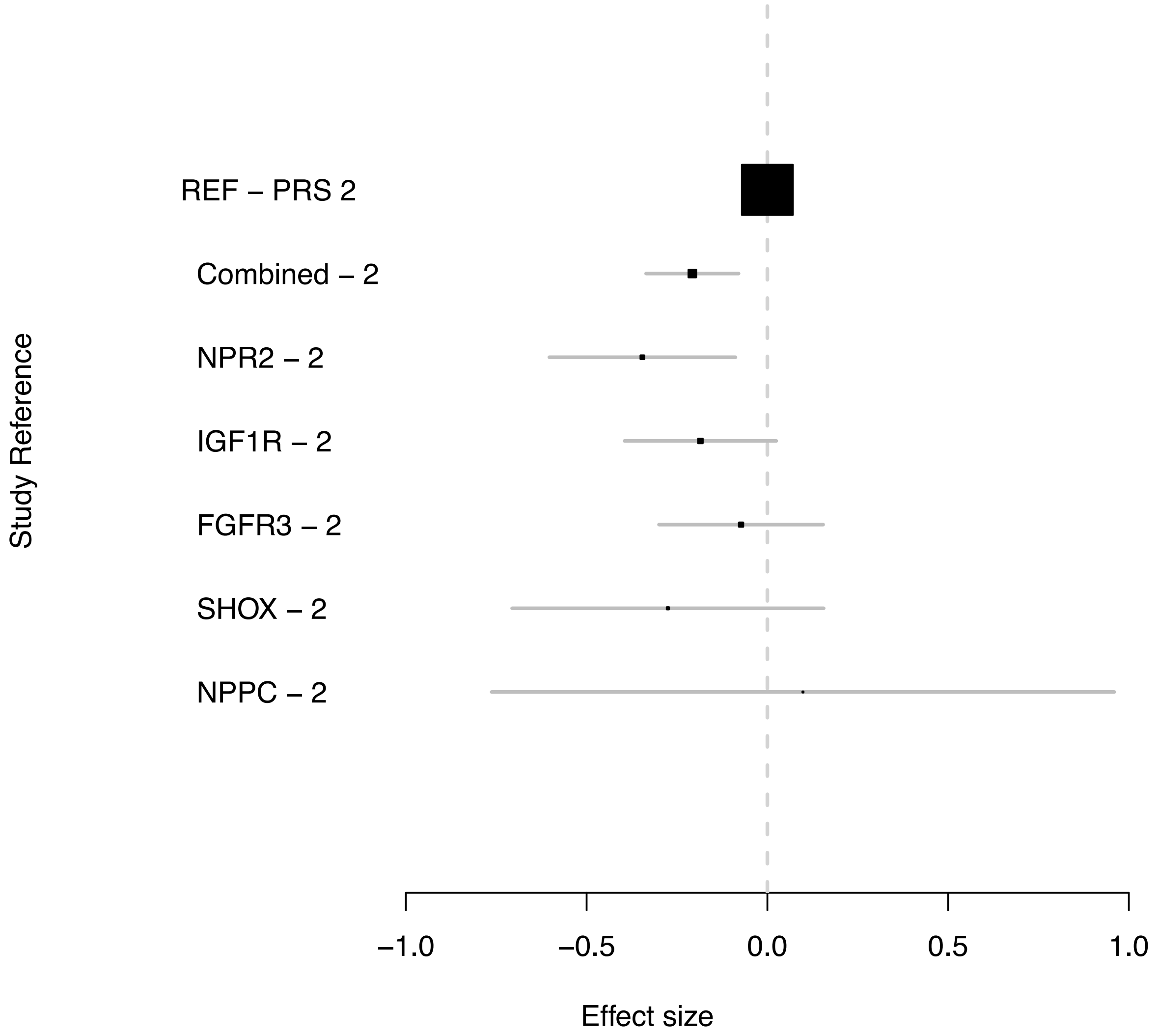

Supplementary Figure 20. Effect of having a protein altering variant in five genes using non-carriers at PRS 2 quintile as reference.

Measure of center for the error bars represent the mean effect. Error bars represent 95% confidence intervals. n=937 samples

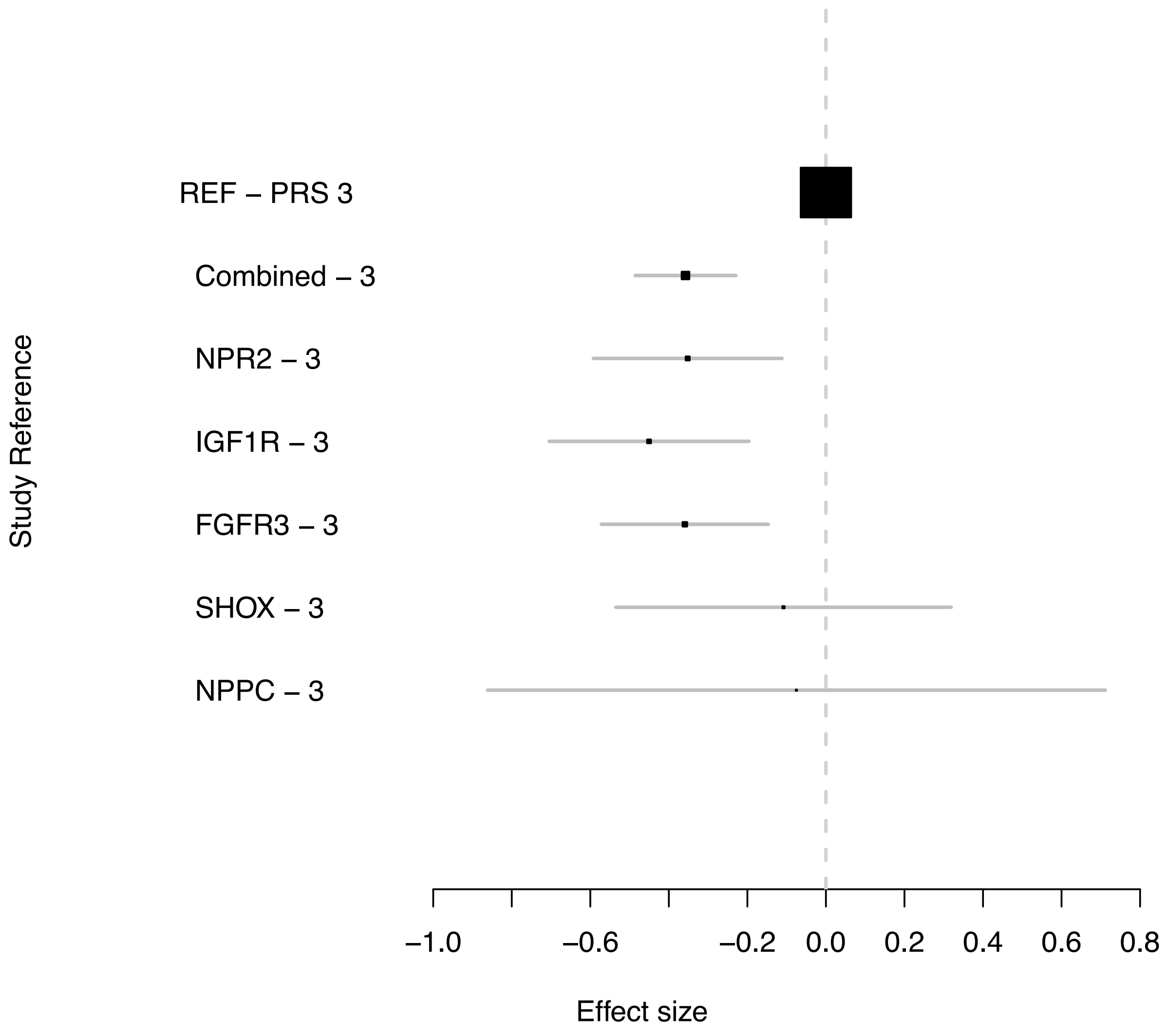

Supplementary Figure 21. Effect of having a protein altering variant in five genes using non-carriers at PRS 3 quintile as reference.

Measure of center for the error bars represent the mean effect. Error bars represent 95% confidence intervals. n=937 samples

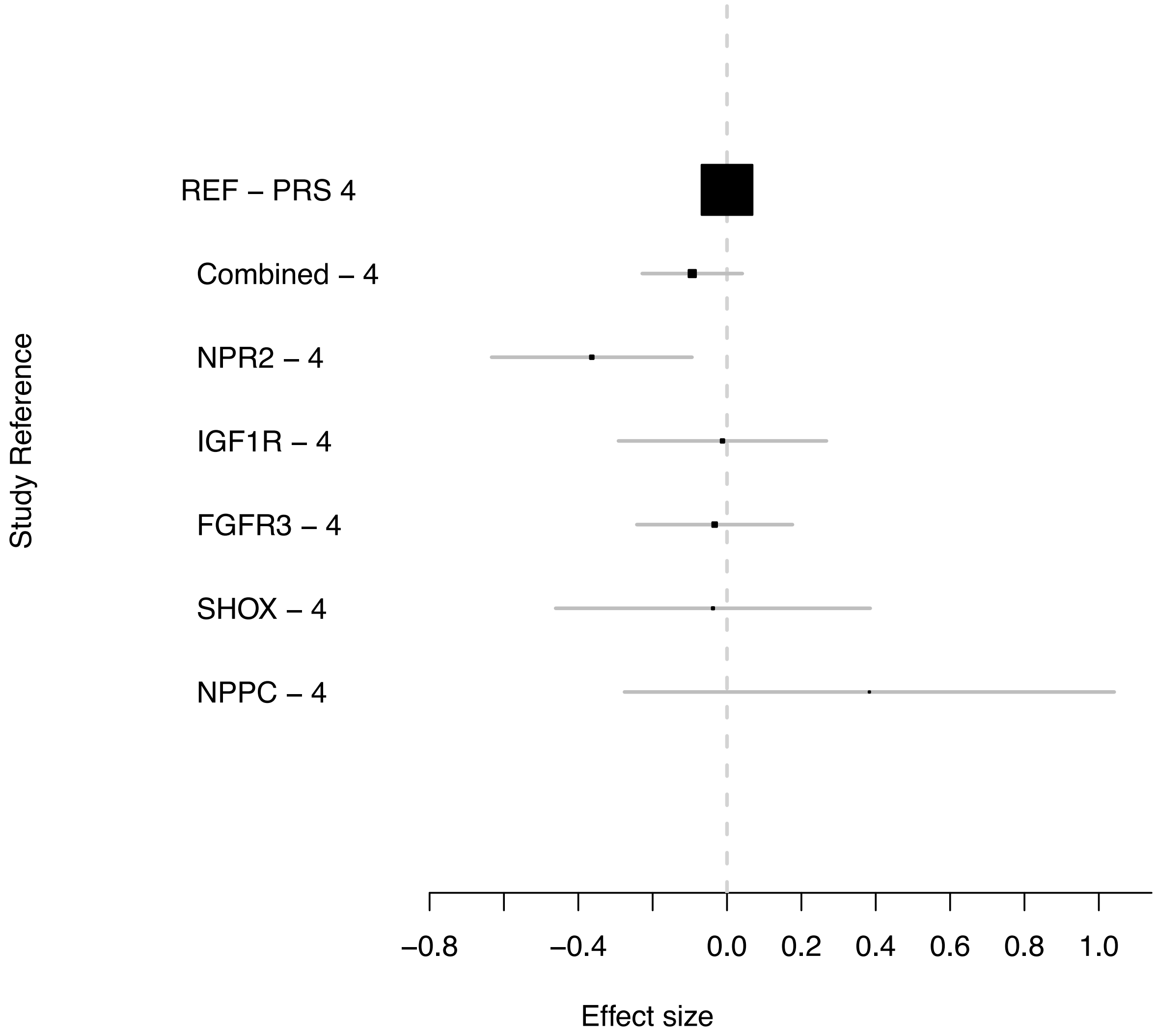

Supplementary Figure 22. Effect of having a protein altering variant in five genes using non-carriers at PRS 4 quintile as reference.

Measure of center for the error bars represent the mean effect. Error bars represent 95% confidence intervals. n=937 samples

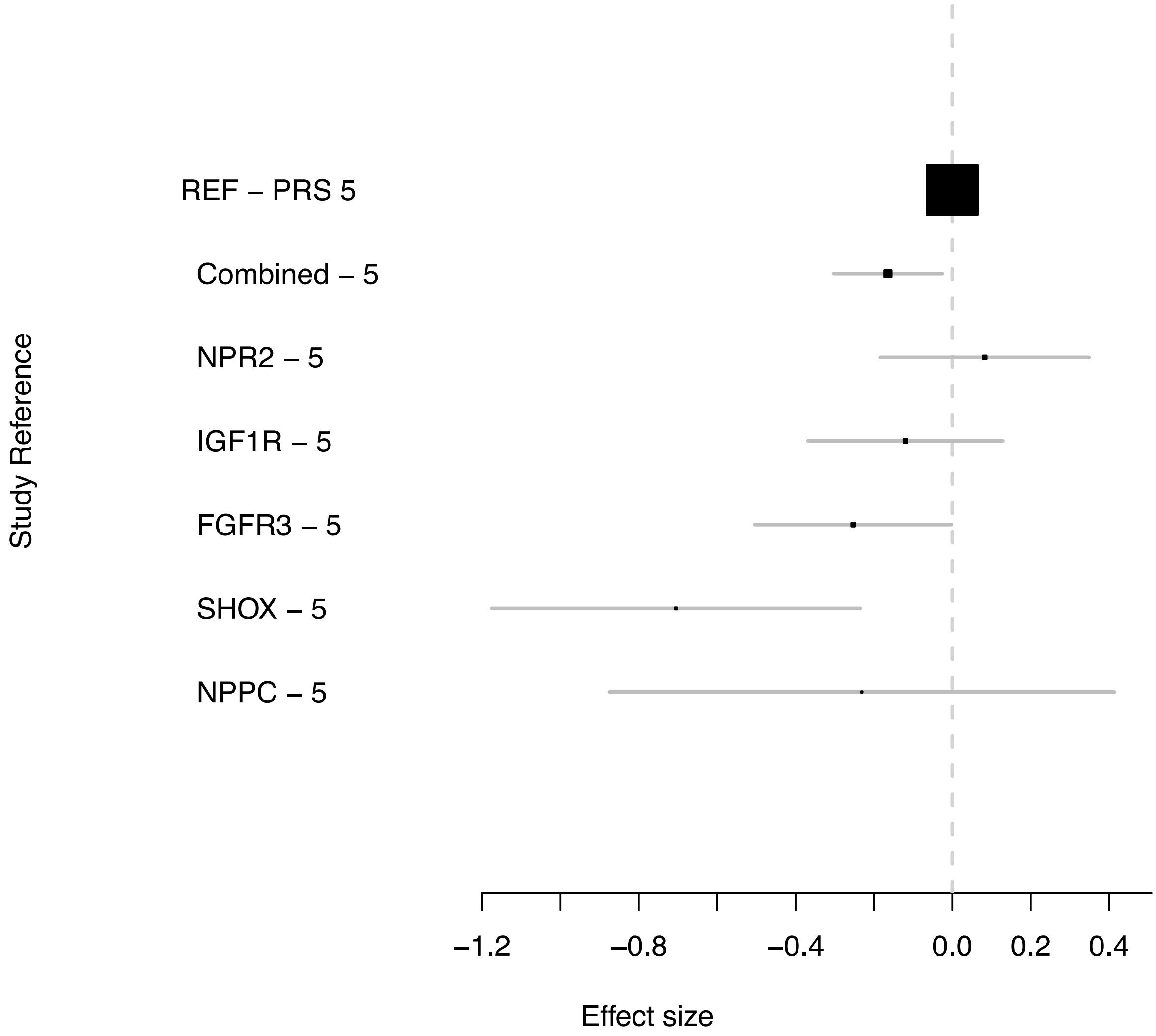

Supplementary Figure 23. Effect of having a protein altering variant in five genes using non-carriers at PRS 5 quintile as reference

Measure of center for the error bars represent the mean effect. Error bars represent 95% confidence intervals. n=937 samples

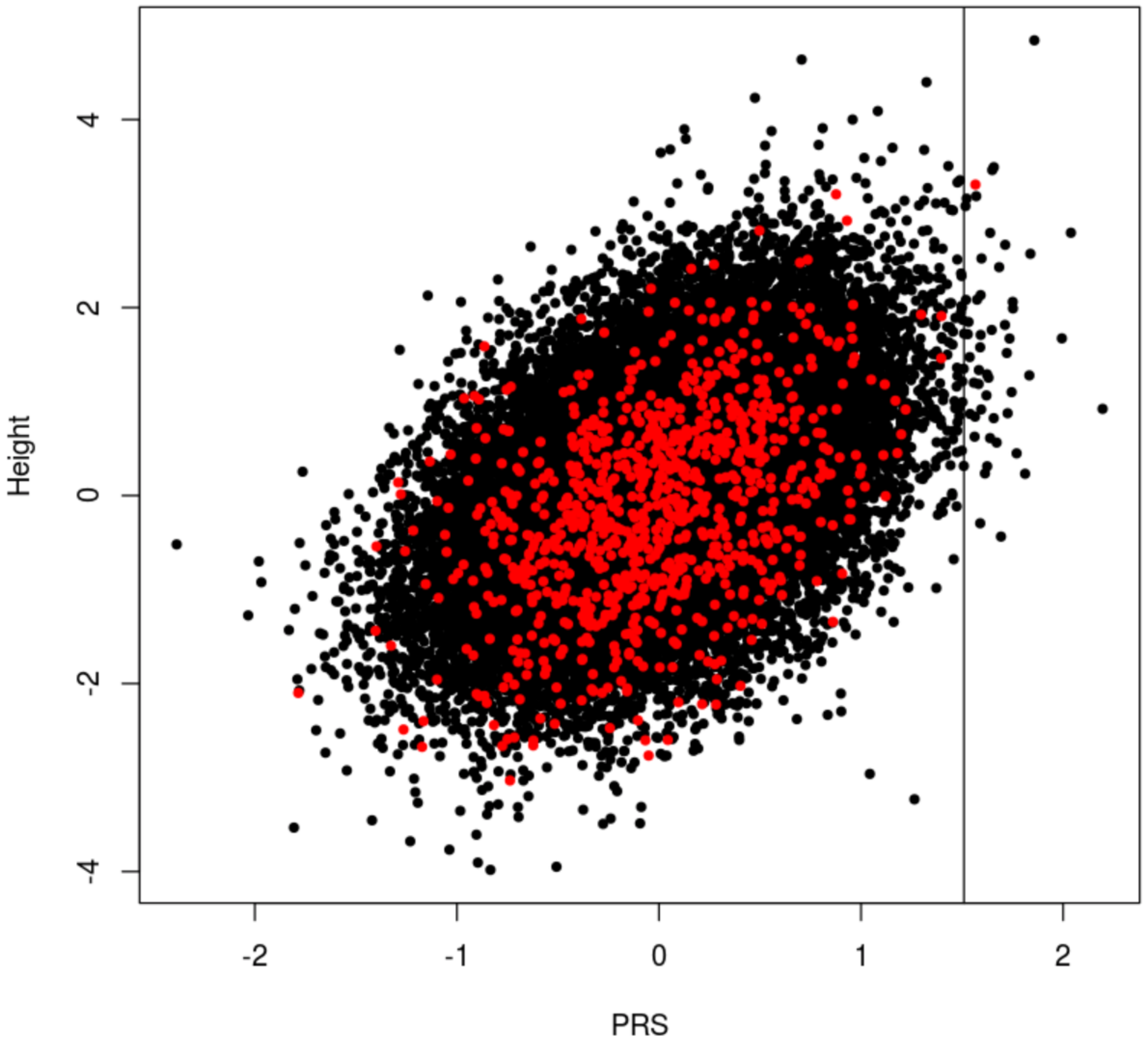

Supplementary Figure 24. Plot of PRS vs Height in all exome sequenced individuals.

Red dots represent carriers of any of the five genes. Black dots are non-carriers. Black vertical line represents 99.85% percentile of the PRS

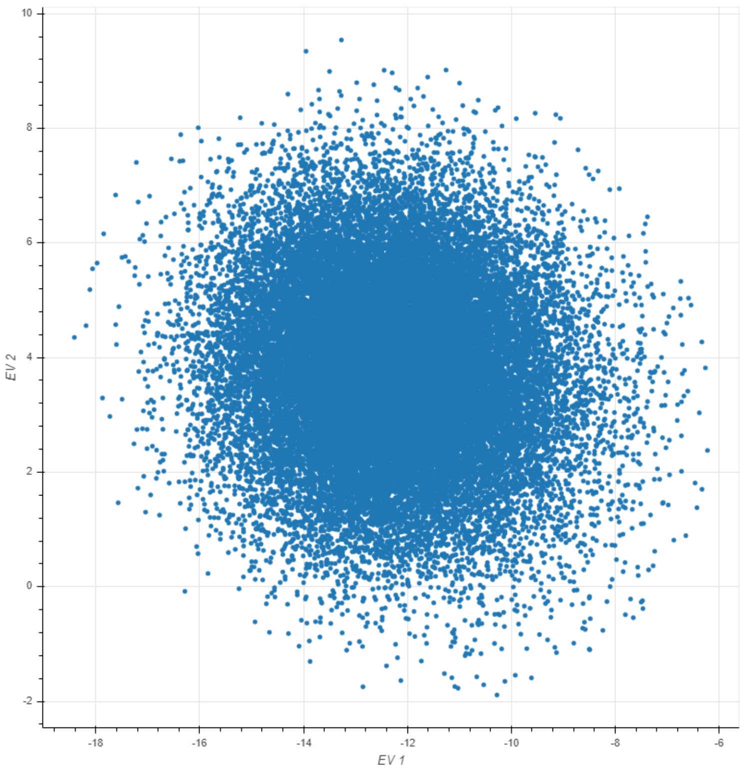

Supplementary Figure 25. Eigenvectors 1 and 2 for 34,284 individuals with exome sequencing passing filters.

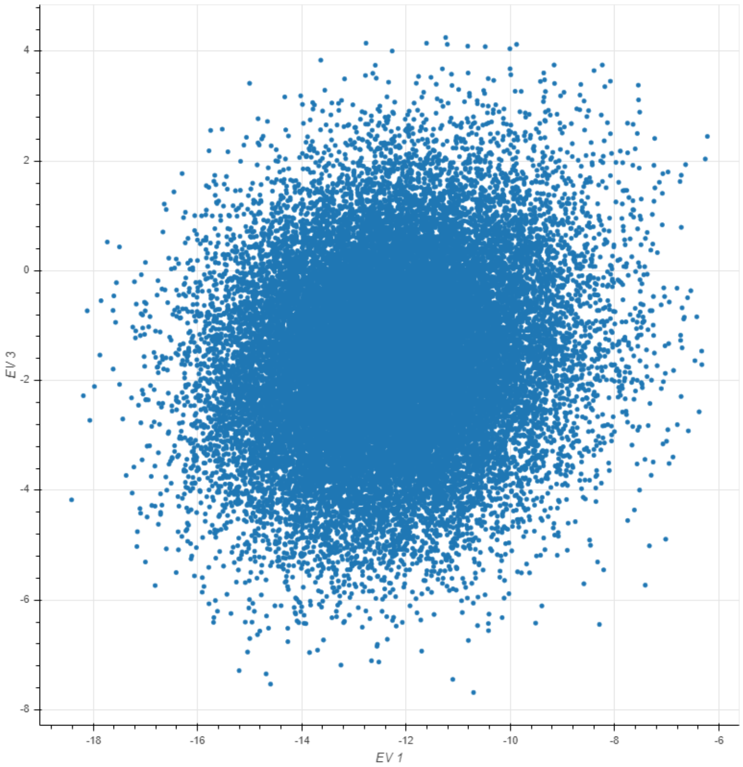

Supplementary Figure 26. Eigenvectors 1 and 3 for 34,284 individuals with exome sequencing passing filters.

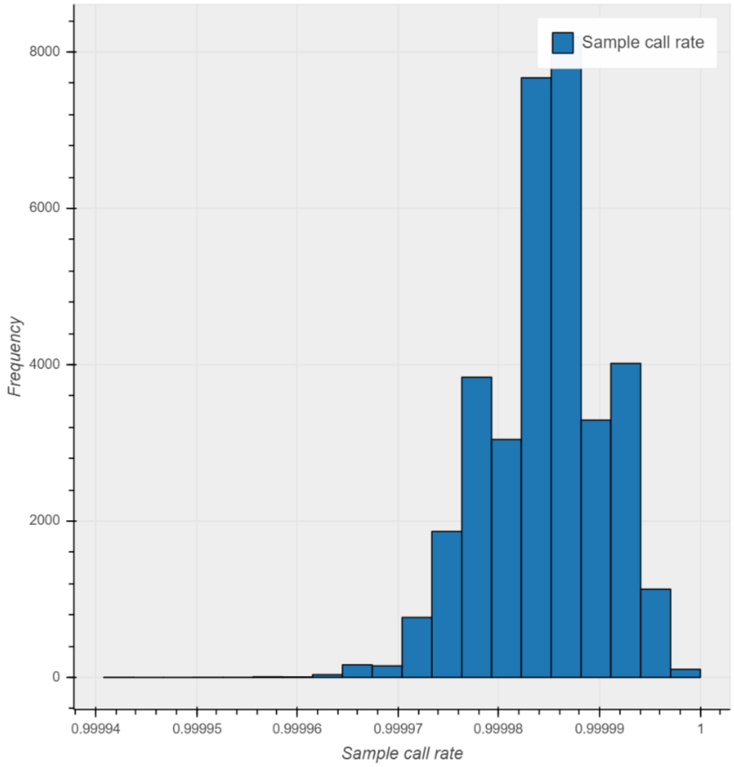

Supplementary Figure 27. Call rate distribution for samples

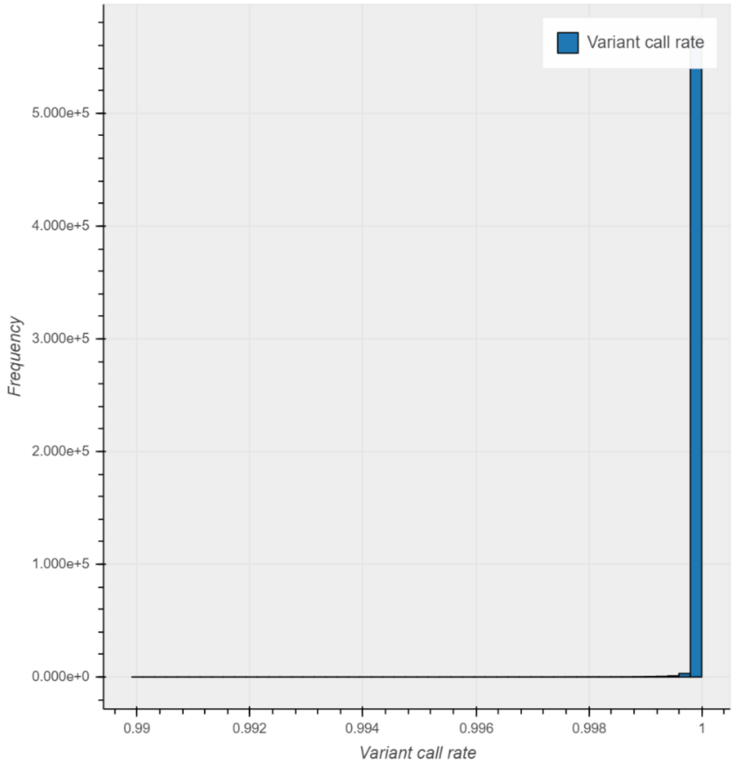

Supplementary Figure 28. Call rate distribution for variants.

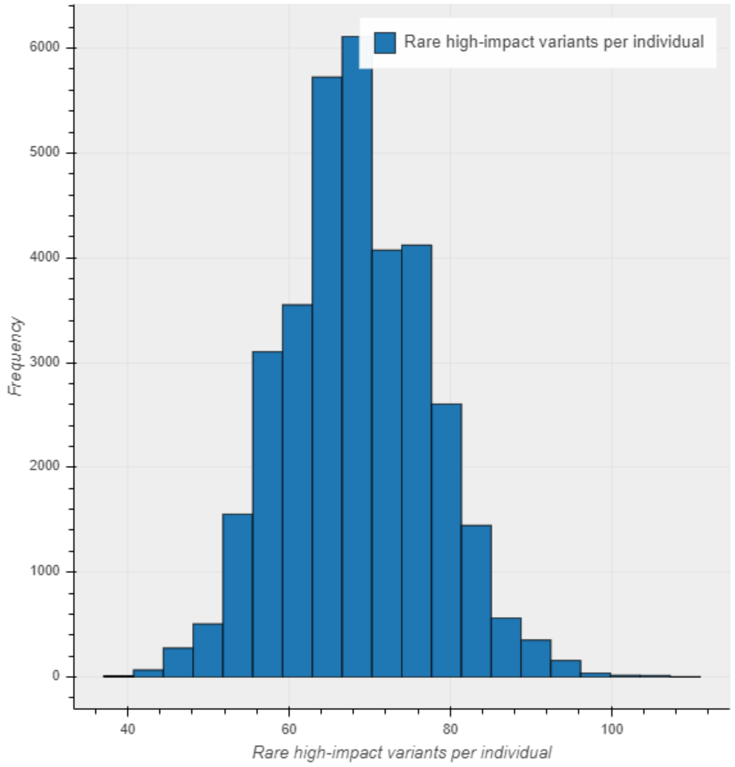

Supplementary Figure 29. Rare high-impact variants per individual

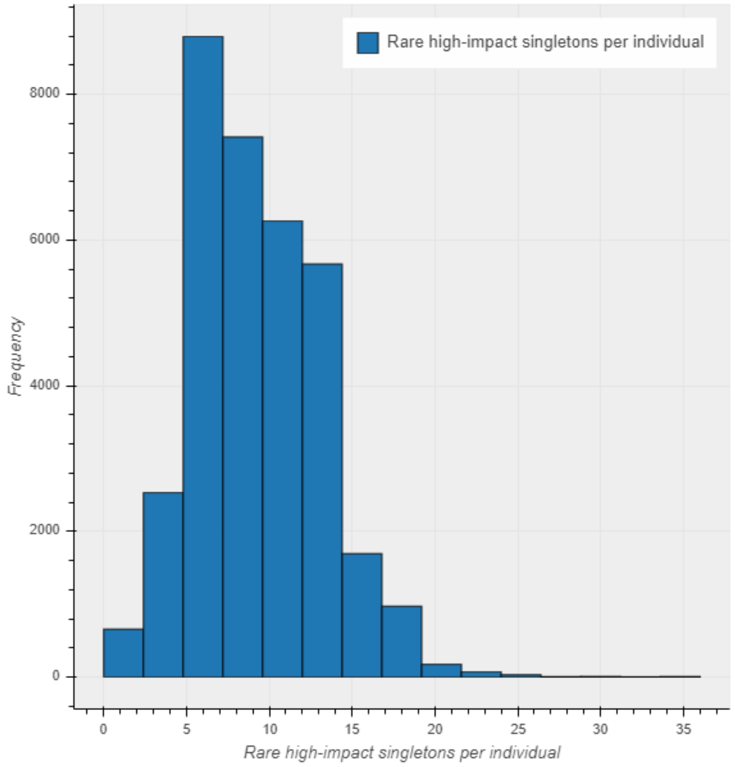

Supplementary Figure 30. Rare high-impact singletons per individual

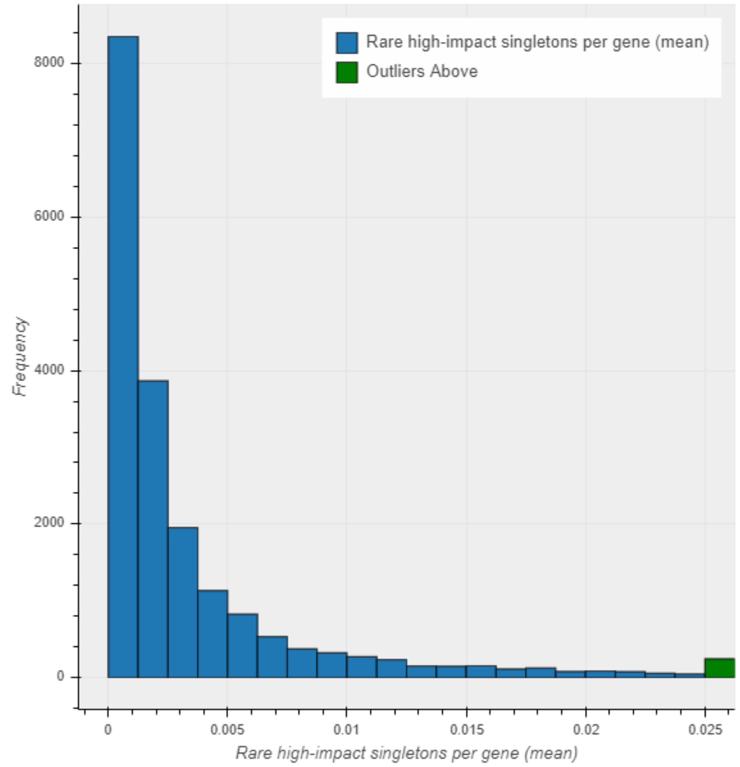

Supplementary Figure 31. Proportion of individuals (out of 34,284) with a rare high-impact variant for each gene (n=19,117)

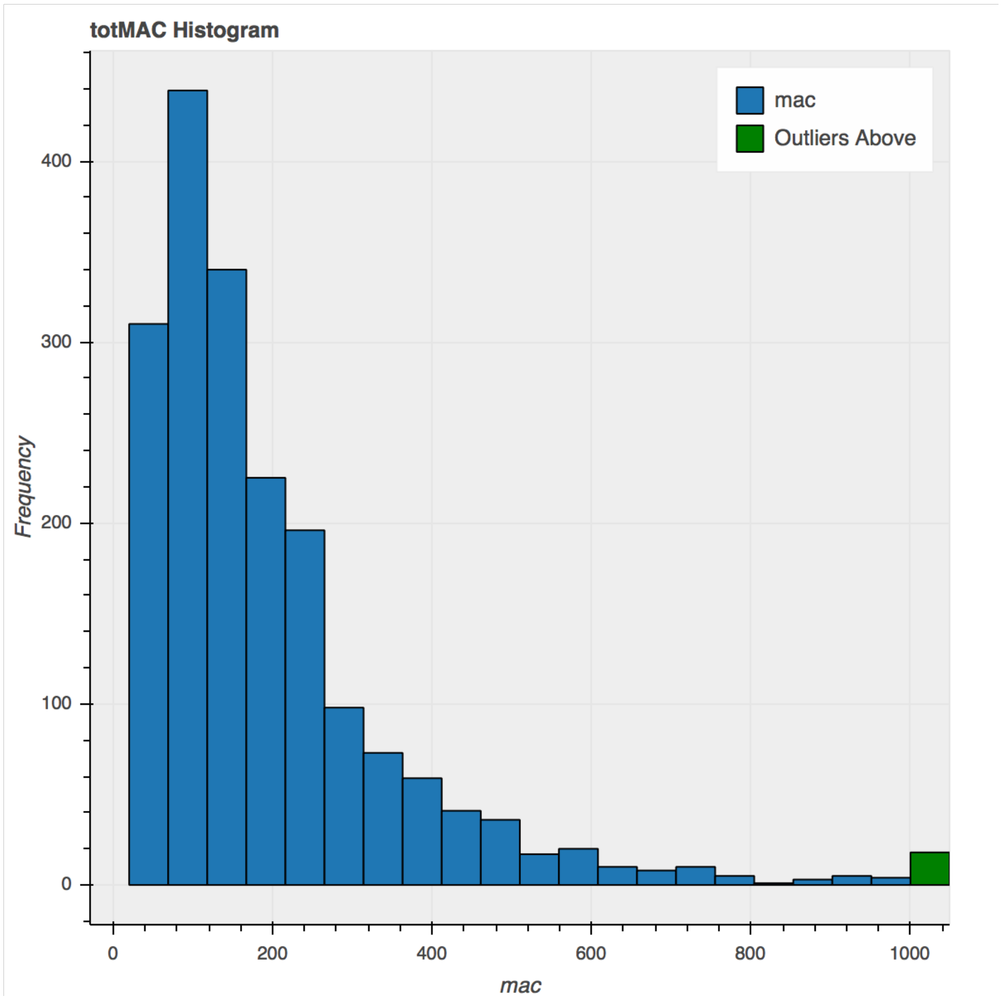

Supplementary Figure 32. Distribution of minor allele counts in the 1918 genes included in gene set

Supplementary Note

Sequence of exon 1 of the NPR2 gene in CRISPR edited clones.

$NPR2^{+/+}$ clone has one allele matching that of wild-type RCS cells. In the $NPR2^{+/-}$ clone allele 1 matches that of wild-type cells and allele 2 has a 2bp insertion. The $NPR2^{-/-}$ clone has a 1bp insertion and allele 2 has a 313bp deletion.

$NPR2^{+/+}$ clone, Allele 1:
ATGGCACTGCCATCCCTGCTACTGGTGGTGGCAGCCCTGGCAGGTGGGGTGCGTCCTCCGGGGGCACGGAACCTGACGCTGGCGGTGGTGC
TGCCAGAACACAACCTGAGCTATGCCTGGGCCTGGCCACGGGTGGGTCCTGCTGTGGCACTGGCTGTGGAGGCGCTGGGCCGGGCACTGCC
CGTGGACCTGCGGTTTGTCAGCTCCGAACTAGACGGCGCCTGCTCTGAGTACCTGGCACCACTGCGCGCTGTGGATCTCAAGCTGTACCATGA
CCCCGACCTTCTGTTGGGCCCTGGTTGTGTGTACCCTGCTGCCTCTGTGGCTCGCTTTGCCTCGCACTGGCACCTTCCCCTCCTGACTGCGGGT
GCAGTGGCCTCTGGCTTTGCAGCTAAGAATGAGCATTATCGTACCCTGGTTCGCACTGGCCCCTCTGCGCCCAAGCTGGGTGAGTTCGTAGTG
ACATTACACGGGCACTTCAATTGGACTGCTCGGGCTGCTTTGCTGTATCTGGATGCTCGCACAGATGACCGGCCCCACTACTTCACCATCGAG
GGGGTGTTTGAGGCCCTGCAGGGCAGCAACCTCAGTGTGCAACACCAGGTGTATACCCGAGAGCCAGGTGGCCCTGAGCAAGCCACCCACT
TCATCAGAGCCAACGGGCGCA

$NPR2^{+/-}$ clone, Allele 1:
ATGGCACTGCCATCCCTGCTACTGGTGGTGGCAGCCCTGGCAGGTGGGGTGCGTCCTCCGGGGGCACGGAACCTGACGCTGGCGGTGGTGC
TGCCAGAACACAACCTGAGCTATGCCTGGGCCTGGCCACGGGTGGGTCCTGCTGTGGCACTGGCTGTGGAGGCGCTGGGCCGGGCACTGCC
CGTGGACCTGCGGTTTGTCAGCTCCGAACTAGACGGCGCCTGCTCTGAGTACCTGGCACCACTGCGCGCTGTGGATCTCAAGCTGTACCATGA
CCCCGACCTTCTGTTGGGCCCTGGTTGTGTGTACCCTGCTGCCTCTGTGGCTCGCTTTGCCTCGCACTGGCACCTTCCCCTCCTGACTGCGGGT
GCAGTGGCCTCTGGCTTTGCAGCTAAGAATGAGCATTATCGTACCCTGGTTCGCACTGGCCCCTCTGCGCCCAAGCTGGGTGAGTTCGTAGTG
ACATTACACGGGCACTTCAATTGGACTGCTCGGGCTGCTTTGCTGTATCTGGATGCTCGCACAGATGACCGGCCCCACTACTTCACCATCGAG
GGGGTGTTTGAGGCCCTGCAGGGCAGCAACCTCAGTGTGCAACACCAGGTGTATACCCGAGAGCCAGGTGGCCCTGAGCAAGCCACCCACT
TCATCAGAGCCAACGGGCGCA

$NPR2^{+/-}$ clone, Allele 2:
ATGGCACTGCCATCCCTGCTACTGGTGGTGGCAGCCCTGGCAGGTGGGGTGCGTCCTCCGGGGGCACGGAACCTGACGCTGGCGGTGGTGC
TGCCAGAACACAACCTGAGCTATGCCTGGGCCTGGCCACGGGTGGGTCCTGCTGTGGCACTGGCTGTGGAGGCGCTGGGCCGGGCACTGCC
CGTGGACCTGCGGTTTGTCAGCTCCGAACTAGACGGCGCCTGCTCTGAGTACCTGGCACCACTGCGCGCTGTGGATCTCAAGCTGTACCATGA
CCCCGACCTTCTGTTGGGCCCTGGTTGTGTGTACCCTGCTGCCTCTGTGGCTCGCTTTGCCTCGCACTGGCACCTTCCCCTCCTGACTGCGGGT
GCAGTGGCCTCTGGCTTTGCAGCTAAGAATGAGCATTATCGTACCCTGGTTCGCACTGGCCCCTCTGCGCCCAAGCTGGGTGAGTTCGTAGTG
ACATTACACGGGCACTTCACAATTGGACTGCTCGGGCTGCTTTGCTGTATCTGGATGCTCGCACAGATGACCGGCCCCACTACTTCACCATCG
AGGGGGTGTTTGAGGCCCTGCAGGGCAGCAACCTCAGTGTGCAACACCAGGTGTATACCCGAGAGCCAGGTGGCCCTGAGCAAGCCACCCA
CTTCATCAGAGCCAACGGGCGCA

$NPR2^{-/-}$ clone, Allele 1:
ATGGCACTGCCATCCCTGCTACTGGTGGTGGCAGCCCTGGCAGGTGGGGTGCGTCCTCCGGGGGCACGGAACCTGACGCTGGCGGTGGTGC
TGCCAGAACACAACCTGAGCTATGCCTGGGCCTGGCCACGGGTGGGTCCTGCTGTGGCACTGGCTGTGGAGGCGCTGGGCCGGGCACTGCC
CGTGGACCTGCGGTTTGTCAGCTCCGAACTAGACGGCGCCTGCTCTGAGTACCTGGCACCACTGCGCGCTGTGGATCTCAAGCTGTACCATGA
CCCCGACCTTCTGTTGGGCCCTGGTTGTGTGTACCCTGCTGCCTCTGTGGCTCGCTTTGCCTCGCACTGGCACCTTCCCCTCCTGACTGCGGGT
GCAGTGGCCTCTGGCTTTGCAGCTAAGAATGAGCATTATCGTACCCTGGTTCGCACTGGCCCCTCTGCGCCCAAGCTGGGTGAGTTCGTAGTG
ACATTACACGGGCACTTCAATTGGACTGCTCGGGCTGCTTTGCTGTATCTGGATGCTCGCACAGATGACCGGCCCCACTACTTCACCATC**C**GA
GGGGGTGTTTGAGGCCCTGCAGGGCAGCAACCTCAGTGTGCAACACCAGGTGTATACCCGAGAGCCAGGTGGCCCTGAGCAAGCCACCCAC
TTCATCAGAGCCAACGGGCGCA

$NPR2^{-/-}$ clone, Allele 2:
ATGGCACTGCCATCCCTGCTACTGGTGGTGGCAGCCCTGGCAGGTGGGGTGCGTCCTCCGGGGGCACGGAACCTGACGCTGGCGGTGGTGC
TGCCAGAACACAACCTGAGCTATGCCTGGGCCTGGCCACGGGTGGGTCCTGCTGTGGCACTGGCTGTGGAGGCGCTGGGCCGGGCACTGCC
CGTGGACCTGCGGTTTGTCAGCTCCGAACTAGACGGCGCCTGCTCTGAGTACCTGGCACCGGGGTGTTTGAGGCCCTGCAGGGCAGCAACCT
CAGTGTGCAACACCAGGTGTATACCCGAGAGCCAGGTGGCCCTGAGCAAGCCACCCACTTCATCAGAGCCAACGGGCGCA

Supplementary Tables

Supplementary Table 1 Study Descriptives for UK Biobank samples with exome-sequence data

**Phenotype**	**Gender**	**n**	**Min.**	**1st Q**	**Median**	**Mean**	**3rd Q**	**Max.**	**ISS**	**% ISS**
	Women	18417	137	159	163	163	167	193	309	1.68%
Height	Men	15814	148	172	176	176.3	181	203	263	1.66%
	Women	18417	40	50	58	56.5	63	70		
Age	Men	15814	40	52	59	57.59	64	70		

Supplementary Table 2 SKAT Association results for the 19 gene sets tested on height

Gene set id	labels	Miss+LoF	Miss	LoF
1	[1, 1, 1, 1, 1]	6.67E-07	2.85E-05	1.27E-05
2	[0, 1, 1, 1, 1]	1.76E-05	5.12E-05	0.05
3	[0, 1, 0, 0, 1]	0.01	0.01	0.41
4	[1, 1, 0, 0, 0]	0.03	0.10	0.01
5	[0, 0, 1, 0, 0]	0.03	0.05	0.33
6	[0, 1, 0, 0, 0]	0.05	0.03	0.70
7	[1, 0, 0, 1, 0]	0.08	0.06	0.48
8	[1, 0, 0, 0, 0]	0.13	0.05	0.06
9	[1, 0, 1, 1, 0]	0.27	0.61	0.03
10	[1, 1, 0, 1, 0]	0.28	0.28	NA
11	[1, 0, 1, 0, 0]	0.42	0.44	0.80
12	[1, 0, 0, 0, 1]	0.44	0.46	0.84
13	[0, 0, 0, 1, 1]	0.53	0.53	1.00
14	[0, 0, 0, 0, 0]	0.68	0.85	0.37
15	[1, 1, 0, 0, 1]	0.69	0.88	0.00
16	[0, 0, 0, 0, 1]	0.70	NA	0.13
17	[0, 0, 0, 1, 0]	0.75	0.89	0.34
18	[1, 0, 1, 1, 1]	0.85	0.73	0.07
19	[1, 0, 0, 1, 1]	0.89	0.92	0.73

Labels represent wether gene is present in annotation (1=present, 0= not present)
Label Order: GWAS, HGMD_Short, HGMD_Tall, OMIM_overgrowth, OMIM_short
Bonferroni significance threashold: 0.003
Only gene sets with at least one gene are reported.

Supplementary Table 3. Proposed mechanisms of identified genes for growth regulation. Lines of evidence from bi-directional genetic effects (gain of function, loss of function) and the associated impact on growth in human, mouse and cell line models.

			Human		Mouse		Cell Line	
**Gene**	**Protein**	**Mechanism**	**GOF**	**LOF**	**GOF**	**LOF**	**GOF**	**LOF**
IGF1R	Insulin-like growth factor 1 receptor	Regulates cell growth and proliferation after activation by IGF1, IGF2, and insulin	↑ [1]	↓ [2]		↓ [3]	↑ [4]	↓ [4]
FGFR3	*Fibroblast growth factor receptor 3*	*Activates kinase (ERK)/mitogen-activated protein kinase (MAPK) pathway. Regulation of chondrocyte proliferation.*	↓ [5]	↑ [6]	↓ [7]	↑ [8]	↓ [9]	
NPPC	C-type natriuretic peptide (CNP)	Promotes endochondral ossification in chondrocytes after binding NPR2.	↑ [10]	↓ [11]	↑ [10]	↓ [12]	↑ [13]	↓ [13]
NPR2	Natriuretic peptide receptor B.	Primary receptor for CNP. Increases guanylyl cyclase activity (cGMP) and counteracts FGFR3 signaling.	↑ [14,15]	↓ [16,17]		↓ [18]	↑ [15]	↓ [17]
SHOX	Short stature homeobox	Multiple. Represses FGFR3 transcription. Up-regulates NPPB. Regulates cell-death.	↑ [19]	↓ [20]		↓ [21]		↓ [22]

Supplementary Table 4 NPR2 mutations tested in functional experiments

Variant	Mean 95%CI	Source	b	ac
G21R	2.78 [2.19 , 3.37]	This_GOF	1.99	1
A48S	0.07 [0.05 , 0.08]	HGMD_ISS	NA	NA
A59T	3.74 [2.69 , 4.78]	This_GOF	1.76	2
V102A	0.11 [0.07 , 0.15]	This_LoF	-2.20	1
R110G	0.7 [0.52 , 0.88]	This_GOF	3.24	1
A164G	0.3 [0.2 , 0.39]	This_Low	-0.21	13
I226V	0.46 [0.41 , 0.5]	This_LoF	-2.02	1
Y250X	0.02 [0.01 , 0.02]	This_LoF	-2.19	1
R263H	1.86 [1.55 , 2.18]	This_GOF	1.25	2
T297M	0.03 [0.01 , 0.05]	HGMD_ADMD	-2.16	2
P301S	0.08 [0 , 0.17]	This_LoF	-2.67	1
R318Q	0.93 [0.79 , 1.06]	This_Neutral	-0.03	7
R358W	1.02 [1 , 1.04]	This_High	0.16	44
E359A	3 [2.65 , 3.34]	This_GOF	1.33	2
I364fs	0.01 [0.01 , 0.02]	HGMD_ADMD	NA	NA
E389D	0.07 [0.05 , 0.09]	HGMD_ISS	NA	NA
E415K	0.47 [0.35 , 0.6]	This_Neutral	-0.08	4
K480N	1.69 [1.54 , 1.84]	This_GOF	1.57	3
A488P	1.27 [0.63 , 1.91]	HGMD_GOF	NA	NA
I494S	0.04 [0 , 0.08]	HGMD_ISS	NA	NA
Q500X	0.02 [0.01 , 0.03]	HGMD_ADMD	NA	NA
H508D	1.74 [1.64 , 1.84]	This_LoF	-2.28	2
G541S	0.53 [0.16 , 0.91]	This_Neutral	0.67	2
A549T	0.71 [0.69 , 0.72]	HGMD_ISS	-0.21	2
R562Q	1.55 [0.83 , 2.28]	HGMD_GOF	NA	NA
R601H	0.48 [0.4 , 0.55]	This_Low	-0.50	14
R601S	0.06 [0.01 , 0.11]	HGMD_ADMD	NA	NA
E609K	1.01 [0.9 , 1.12]	This_Low	-0.47	13
S721R	0.96 [0.76 , 1.16]	This_Neutral	-0.04	3
E727Q	1.7 [1.53 , 1.87]	This_GOF	1.35	1
R745Q	0.63 [0.44 , 0.82]	This_Neutral	0.02	3
R819C	0.14 [0.11 , 0.17]	HGMD_ISS	-0.58	1
T861I	0.08 [0.06 , 0.1]	This_LoF	-2.12	1
V883M	0.33 [0.26 , 0.41]	HGMD_GOF	NA	NA
T907M	0.01 [0.01 , 0.02]	HGMD_ADMD	NA	NA
G917R	0.02 [0.01 , 0.02]	This_LoF	-2.74	1
R921Q	0.22 [0.2 , 0.25]	This_LoF	-1.91	1
R957C	0.03 [0.03 , 0.03]	HGMD_ADMD	-0.86	1
L1009V	0.08 [0.07 , 0.09]	This_LoF	-2.28	2
Wild-Type	1 [0.91 , 1.09]			

Abreviations: source, Source for choosing this variant for the assay; b, beta estimate for height in the ukbiobank data; AC, allele count in the ukbiobank data

Supplementary Table 5 Median height of PRS x cGMP category in NPR2 carriers

PRS group	cGMP group	n	ZPRS	cGMP	zheight
1	(0.0, 0.2]	3	-1.38	0.08	-2.60
1	(0.2, 0.5]	8	-1.48	0.47	-1.17
1	(0.5, 1.5]	14	-1.00	1.02	-0.56
1	(1.5, 4.0]	0	NA	NA	NA
2	(0.0, 0.2]	1	-0.63	0.03	-2.10
2	(0.2, 0.5]	2	-0.51	0.39	-0.38
2	(0.5, 1.5]	20	-0.47	1.01	-0.06
2	(1.5, 4.0]	1	-0.77	2.78	2.05
3	(0.0, 0.2]	2	0.07	0.05	-2.09
3	(0.2, 0.5]	9	-0.03	0.48	-0.45
3	(0.5, 1.5]	13	0.12	1.01	-0.05
3	(1.5, 4.0]	0	NA	NA	NA
4	(0.0, 0.2]	1	0.73	0.03	-0.80
4	(0.2, 0.5]	8	0.53	0.30	-0.21
4	(0.5, 1.5]	11	0.59	1.02	0.29
4	(1.5, 4.0]	4	0.73	2.43	1.75
5	(0.0, 0.2]	1	1.32	0.14	-0.52
5	(0.2, 0.5]	6	1.43	0.38	0.31
5	(0.5, 1.5]	14	1.38	1.02	0.38
5	(1.5, 4.0]	4	1.91	1.72	1.12

Abreviations: n, number of individuals in group; ZPRS,Median of Polygenic risk score in group; cGMP,median of cyclic GMP in group; zheight, median of height in group

References

1. Kant, S.G. *et al.* Tall stature and duplication of the insulin-like growth factor I receptor gene. *Eur J Med Genet* **50**, 1-10 (2007).
2. Keselman, A.C. *et al.* A Homozygous Mutation in the Highly Conserved Tyr60 of the Mature IGF1 Peptide Broadens the Spectrum of IGF1 Deficiency. *Eur J Endocrinol* (2019).
3. Liu, J.P., Baker, J., Perkins, A.S., Robertson, E.J. & Efstratiadis, A. Mice carrying null mutations of the genes encoding insulin-like growth factor I (Igf-1) and type 1 IGF receptor (Igf1r). *Cell* **75**, 59-72 (1993).
4. Okubo, Y. *et al.* Cell Proliferation Activities on Skin Fibroblasts from a Short Child with Absence of One Copy of the Type 1 Insulin-Like Growth Factor Receptor (IGF1R) Gene and a Tall Child with Three Copies of the IGF1R Gene. *The Journal of Clinical Endocrinology & Metabolism* **88**, 5981-5988 (2003).
5. Shiang, R. *et al.* Mutations in the transmembrane domain of FGFR3 cause the most common genetic form of dwarfism, achondroplasia. *Cell* **78**, 335-42 (1994).
6. Toydemir, R.M. *et al.* A novel mutation in FGFR3 causes camptodactyly, tall stature, and hearing loss (CATSHL) syndrome. *Am J Hum Genet* **79**, 935-41 (2006).
7. Yasoda, A. *et al.* Overexpression of CNP in chondrocytes rescues achondroplasia through a MAPK-dependent pathway. *Nat Med* **10**, 80-6 (2004).
8. Colvin, J.S., Bohne, B.A., Harding, G.W., McEwen, D.G. & Ornitz, D.M. Skeletal overgrowth and deafness in mice lacking fibroblast growth factor receptor 3. *Nat Genet* **12**, 390-7 (1996).
9. Krejci, P. *et al.* Analysis of STAT1 activation by six FGFR3 mutants associated with skeletal dysplasia undermines dominant role of STAT1 in FGFR3 signaling in cartilage. *PLoS One* **3**, e3961 (2008).
10. Bocciardi, R. *et al.* Overexpression of the C-type natriuretic peptide (CNP) is associated with overgrowth and bone anomalies in an individual with balanced t(2;7) translocation. *Hum Mutat* **28**, 724-31 (2007).
11. Tassano, E. *et al.* Genotype-Phenotype Correlation of 2q37 Deletions Including NPPC Gene Associated with Skeletal Malformations. *PLoS One* **8**, e66048 (2013).
12. Chusho, H. *et al.* Dwarfism and early death in mice lacking C-type natriuretic peptide. *Proc Natl Acad Sci U S A* **98**, 4016-21 (2001).
13. Peake, N.J. *et al.* Role of C-type natriuretic peptide signalling in maintaining cartilage and bone function. *Osteoarthritis Cartilage* **22**, 1800-7 (2014).
14. Miura, K. *et al.* Overgrowth syndrome associated with a gain-of-function mutation of the natriuretic peptide receptor 2 (NPR2) gene. *Am J Med Genet A* **164A**, 156-63 (2014).
15. Miura, K. *et al.* An overgrowth disorder associated with excessive production of cGMP due to a gain-of-function mutation of the natriuretic peptide receptor 2 gene. *PLoS One* **7**, e42180 (2012).
16. Bartels, C.F. *et al.* Mutations in the transmembrane natriuretic peptide receptor NPR-B impair skeletal growth and cause acromesomelic dysplasia, type Maroteaux. *Am J Hum Genet* **75**, 27-34 (2004).
17. Wang, S.R. *et al.* Heterozygous mutations in natriuretic peptide receptor-B (NPR2) gene as a cause of short stature. *Hum Mutat* **36**, 474-81 (2015).

18. Tsuji, T. & Kunieda, T. A loss-of-function mutation in natriuretic peptide receptor 2 (Npr2) gene is responsible for disproportionate dwarfism in cn/cn mouse. *J Biol Chem* **280**, 14288-92 (2005).
19. Ogata, T. *et al.* Short stature homeobox-containing gene duplication on the der(X) chromosome in a female with 45,X/46,X, der(X), gonadal dysgenesis, and tall stature. *J Clin Endocrinol Metab* **85**, 2927-30 (2000).
20. Rao, E. *et al.* Pseudoautosomal deletions encompassing a novel homeobox gene cause growth failure in idiopathic short stature and Turner syndrome. *Nat Genet* **16**, 54-63 (1997).
21. Burgoyne, P.S., Ojarikre, O.A. & Turner, J.M. Evidence that postnatal growth retardation in XO mice is due to haploinsufficiency for a non-PAR X gene. *Cytogenet Genome Res* **99**, 252-6 (2002).
22. Hristov, G. *et al.* SHOX triggers the lysosomal pathway of apoptosis via oxidative stress. **23**, 1619-1630 (2014).
