# Supplementary material for: Identifying therapeutic drug targets using bidirectional effect genes
Source: Nat Commun. 2021 Apr 13;12:2224. doi: 10.1038/s41467-021-21843-8 (PMC8044152; doi:10.1038/s41467-021-21843-8)
Supplement: Supplementary file 9 — Reporting Summary [file 41467_2021_21843_MOESM9_ESM.pdf]

nature research

Corresponding author(s): Karol Estrada

Last updated by author(s): 2021-01-11

Reporting Summary

Nature Research wishes to improve the reproducibility of the work that we publish. This form provides structure for consistency and transparency in reporting. For further information on Nature Research policies, see our Editorial Policies and the Editorial Policy Checklist.

Statistics

For all statistical analyses, confirm that the following items are present in the figure legend, table legend, main text, or Methods section.

n/a	Confirmed	
☐	☒	The exact sample size (*n*) for each experimental group/condition, given as a discrete number and unit of measurement
☐	☒	A statement on whether measurements were taken from distinct samples or whether the same sample was measured repeatedly
☐	☒	The statistical test(s) used AND whether they are one- or two-sided<br>*Only common tests should be described solely by name; describe more complex techniques in the Methods section.*
☐	☒	A description of all covariates tested
☐	☒	A description of any assumptions or corrections, such as tests of normality and adjustment for multiple comparisons
☐	☒	A full description of the statistical parameters including central tendency (e.g. means) or other basic estimates (e.g. regression coefficient) AND variation (e.g. standard deviation) or associated estimates of uncertainty (e.g. confidence intervals)
☐	☒	For null hypothesis testing, the test statistic (e.g. *F*, *t*, *r*) with confidence intervals, effect sizes, degrees of freedom and *P* value noted<br>*Give P values as exact values whenever suitable.*
☒	☐	For Bayesian analysis, information on the choice of priors and Markov chain Monte Carlo settings
☒	☐	For hierarchical and complex designs, identification of the appropriate level for tests and full reporting of outcomes
☐	☒	Estimates of effect sizes (e.g. Cohen's *d*, Pearson's *r*), indicating how they were calculated

Our web collection on statistics for biologists contains articles on many of the points above.

Software and code

Policy information about availability of computer code

Data collection	Genetic data collection was performed on Hail 0.2.16 running on Apache Spark version 2.3.4.
Data analysis	Genetic data analysis: Hail 0.2.16 / Apache Spark v2.3.4. Sequence: SMRTlink 6.0 software. Geneious Prime 2019.0.4. R/3.5.2 and R v3.6.1 , LDpred v1.0.7 , PAGEANT v1. Variant Effect Predictor release 96,

All software used for this paper is publicly available. The code used is available upon request.

Data

Policy information about availability of data

All manuscripts must include a data availability statement. This statement should provide the following information, where applicable:

- Accession codes, unique identifiers, or web links for publicly available datasets
- A list of figures that have associated raw data
- A description of any restrictions on data availability

Data used for this research is available on their respective public repositories.
The UK Biobank exome sequence data was obtained under application 41232. These data are available to approved researchers through the UKB Data Showcase at http://ukbiobank.ac.uk/
Full table of genetic evidence and approved drugs is available at: https://github.com/AbbVie-ComputationalGenomics/genetic-evidence-approval.

Field-specific reporting

Life sciences study design

All studies must disclose on these points even when the disclosure is negative.

Sample size

We determined that with the current sample size (34, 284) our study had 63% of power to identify a gene set (group of genes) that would explain 0.09% of the variance on height. We used PAGEANT to estimate the power using an alpha=0.002 (adjusting for 25 gene set tests).

Data exclusions

As we were interested in bidirectional genes, any gene in HGMD with less than two unique disease associations were excluded from further analysis. To focus on disease relevant genes, associations between genes and molecular, cellular, or benign phenotypes (e.g. increased and decreased enzyme activity, cell permeability, or pigmentation) were also excluded.

We applied a number of pre-specified sample and variant QC in the UKBiobank data. These filters were implemented to identify samples and sites that have significant evidence of random noise. This is performed by removing any sample or variant that significantly deviate from the rest on key QC parameters.

We annotated and filtered individuals with data provided by UK Biobank. Specifically, we included individuals with the genetic ethnic grouping “Caucasian” (field 2206), no sex chromosome aneuploidies (field 22019) (effectively removing any classical Turner syndrome patients) and with a PCA-corrected heterozygosity between 0.17 and 0.21 (field 22019). After applying these filters, our dataset contained 41,190 individuals.. we removed related individuals by excluding samples meeting the criteria for relatedness and kinship set by UK Biobank (2nd degree relatives or closer) and that are listed in fields 22011, 22012 and 22013.

Additional sample and variant QC were applied before association analyses:
We computed sample and variant QC metrics and applied additional filters that excluded sample with more than 80,000 non-reference variants, more than 200 singletons, a het/hom ration of less than 1.3 or more than 1.85, and transition/transversion ratio of more than 2.5, a call rate of less than 0.985 or a proportion of heterozygous sites in sequencing data that are also heterozygous in chip data of less than 0.98. We also excluded variants that had a call rate of less than 0.99 or a Hardy-Weinberg p value of less than 10^-10.

Replication

For the initial detection of bidirectional genes, we used OMIM records one single time as successful replication of findings from the HGMD database.

Randomization

This is not a randomized study. We adjusted height for age and first five principal components.

Blinding

This is not a randomized blinded study, in genetic studies we don't performed blinding of data. All the data used in this study had been generated by others.

Reporting for specific materials, systems and methods

We require information from authors about some types of materials, experimental systems and methods used in many studies. Here, indicate whether each material, system or method listed is relevant to your study. If you are not sure if a list item applies to your research, read the appropriate section before selecting a response.

Materials & experimental systems

n/a	Involved in the study	
☒	☐	Antibodies
☐	☒	Eukaryotic cell lines
☒	☐	Palaeontology and archaeology
☒	☐	Animals and other organisms
☐	☒	Human research participants
☒	☐	Clinical data
☒	☐	Dual use research of concern

Methods

n/a	Involved in the study	
☒	☐	ChIP-seq
☒	☐	Flow cytometry
☒	☐	MRI-based neuroimaging

Eukaryotic cell lines

Policy information about cell lines

Cell line source(s)	HEK293T (ATCC (Cat. #CRL-3216)), RCS (gift from Pavel Krejčí)
Authentication	No authentication was performed.
Mycoplasma contamination	Cell lines were not tested for mycoplasma contamination
Commonly misidentified lines (See ICLAC register)	There are no commonly misidentified cell lines used in this study.

Human research participants

Policy information about studies involving human research participants

Population characteristics	The demographics and patient characteristics for the 50,000 exome sequenced individuals has been previously described.54 In short, individuals from the UK aged 45 to 75 were invited to participate. A baseline questionnaire and several measurements were taken. Electronic health records were also available. Standing height (field id 50) was measured to all individuals as part of the baseline assessment (Supplementary Table 3). Standing height was adjusted for age and sex and residuals were normalized.
Recruitment	UK Biobank recruited 500,000 people aged between 40-69 years in 2006-2010 from across the country to take part in this project. It has been reported that individuals enrolled in this study are slightly more healthy than the general UK population. It is unclear how this could affect recruitment of short stature individuals, but most likely it would mean that the estimates of prevalence reported here would be a lower bound of the real estimate. No additional self-selection bias were detected.
Ethics oversight	UK Biobank has approval from the North West Multi-centre Research Ethics Committee (MREC), which covers the UK.

Note that full information on the approval of the study protocol must also be provided in the manuscript.
