# Supplementary material for: Identifying therapeutic drug targets using bidirectional effect genes
Source: Nat Commun. 2021 Apr 13;12:2224. doi: 10.1038/s41467-021-21843-8 (PMC8044152; doi:10.1038/s41467-021-21843-8)
Supplement: Supplementary file 10 — Description of Additional Supplementary Files [file 41467_2021_21843_MOESM10_ESM.pdf]

Description of Additional Supplementary Data files

File Name: Supplementary Data 1.

Description: List of potential bidirectional effect selected tagets from HGMD.

File Name: Supplementary Data 2.

Description: List of target - indication pairs with clinical trial results.

File Name: Supplementary Data 3.

Description: Comparison of genetic evidence risk ratio in Phase I to Approval.

File Name: Supplementary Data 4.

Description: List of lead SNPs from GWAS meta-analysis of height (Yengo, 2018).

File Name: Supplementary Data 5.

Description: List of height genes and their respective annotations.

File Name: Supplementary Data 6.

Description: Genetic variants found in five genes.

File Name: Supplementary Data 7.

Description: Number of mutations described in HGMD for each of the five height genes.
